# Charge Transport Systems with Fermi–Dirac Statistics for Memristors

**DOI:** 10.1007/s00332-025-10140-z

**Published:** 2025-02-18

**Authors:** Maxime Herda, Ansgar Jüngel, Stefan Portisch

**Affiliations:** 1 Inria, CNRS, UMR 8524 - Laboratoire Paul Painlevé, University of Lille, 59000 Lille, France; 2 Institute of Analysis and Scientific Computing, TU Wien, Wiedner Hauptstraße 8–10, 1040 Wien, Austria

**Keywords:** Drift–diffusion equations, Fermi–Dirac statistics, Blakemore statistics, Global existence, Bounded weak solutions, Memristors, Semiconductors, Neuromorphic computing, 35B45, 35B65, 35K51, 35K65, 35Q81

## Abstract

An instationary drift–diffusion system for the electron, hole, and oxygen vacancy densities, coupled to the Poisson equation for the electric potential, is analyzed in a bounded domain with mixed Dirichlet–Neumann boundary conditions. The electron and hole densities are governed by Fermi–Dirac statistics, while the oxygen vacancy density is governed by Blakemore statistics. The equations model the charge carrier dynamics in memristive devices used in semiconductor technology. The global existence of weak solutions is proved in up to three space dimensions. The proof is based on the free energy inequality, an iteration argument to improve the integrability of the densities, and estimations of the Fermi–Dirac integral. Under a physically realistic elliptic regularity condition, it is proved that the densities are bounded.

## Introduction

Memristors are nonlinear resistors with memory able to exhibit a resistive switching behavior. In neuromorphic computing, they are used to build artificial neurons and synapses (Ielmini and Ambrogio 2020). Also perovskite solar cells may show a memristive behavior, emulating synaptic- and neural-like dynamics (Tessler and Vaynzof 2020). In semiconductor technology, often oxide-based memristors are used. They consist of a thin titanium dioxide layer between two metal electrodes (Mladenov 2019). Charge carriers are the electrons, holes (defect electrons), and oxide vacancies, which allow for a modulation of the layer conductance. The transport of the carriers is usually modeled by drift–diffusion equations; see Ahmadi et al. (2021), Greenlee et al. (2013)) for memristors and Abdel et al. (2021) for perovskite solar cells.

Generally, the relation between the electron density and its chemical potential (quasi-Fermi potential) is given by Fermi–Dirac statistics. In low-density regimes, this reduces to Maxwell–Boltzmann statistics, leading to particle fluxes with linear diffusion (Jüngel 2009), while in high-density regimes, Fermi–Dirac statistics reduce to a power-law density–chemical potential relation, leading to fluxes with degenerate diffusion. A mathematical analysis of the associated low-density drift–diffusion equations was performed in Jourdana et al. (2023), while high-density models were studied in Jüngel and Vetter (2025). In this paper, we investigate for the first time a general drift–diffusion system with Fermi–Dirac statistics for the electrons and holes as well as physically motivated Blakemore statistics for the oxide vacancies.

### Model Equations

The charge transport through the semiconductor device is supposed to be governed by the mass balance equations for the electron density *n*(*x*, *t*), hole density *p*(*x*, *t*), and density *D*(*x*, *t*) of oxide vacancies, and the gradients of the associated chemical potentials (quasi-Fermi potentials) $\mu _n$, $\mu _p$, and $\mu _D$ are the driving forces of the flow. This leads to the (scaled) equations

$$\begin{aligned} \partial _t n - \operatorname {div}J_n = 0,&\quad J_n = n\nabla \mu _n, \\ \partial _t p + \operatorname {div}J_p = 0,&\quad J_p = -p\nabla \mu _p, \\ \partial _t D + \operatorname {div}J_D = 0,&\quad J_D = -D\nabla \mu _D, \end{aligned}$$

where $J_n$, $J_p$, and $J_D$ are the electron, hole, and oxide vacancy current densities, respectively. Fermi–Dirac statistics is valid for electrons in the conduction band and for holes in the valance band in the parabolic band approximation (Jüngel 2009, Sec. 1.6), giving the relations

$$\begin{aligned} n = \mathcal {F}_{1/2}(\mu _n+V), \quad p = \mathcal {F}_{1/2}(\mu _p-V), \end{aligned}$$

where *V* denotes the electric potential, and the Fermi–Dirac integral is defined by:

$$\begin{aligned} \mathcal {F}_{1/2}(y) = \frac{2}{\sqrt{\pi }}\int _0^\infty \frac{\sqrt{s}}{1+e^{s-y}}\textrm{d}s, \quad y\in {{\mathbb {R}}}. \end{aligned}$$

In the Maxwell–Boltzmann approximation, the Fermi–Dirac integral can be approximated by the exponential, $\mathcal {F}_{1/2}(y)\approx \exp (y)$ for $y\ll -1$, leading to the electron flux $J_n\approx n\nabla (\log n-V)=\nabla n-n\nabla V$. However, the use of Fermi–Dirac statistics is more appropriate in regimes with moderate or high densities. We expect that the oxide vacancies cannot be accumulated excessively such that it is reasonable to use Blakemore statistics (Blakemore 1982),

$$\begin{aligned} D = \mathcal {F}_{-1}(\mu _D-V),\quad \text{ where } \mathcal {F}_{-1}(y) = \frac{1}{1+e^{-y}}, \ y\in {{\mathbb {R}}}. \end{aligned}$$

Although being itself an approximation of Fermi–Dirac statistics, Blackmore statistics have the advantage of restricting the oxide vacancy density to the interval (0, 1). Without loss of generality, we have set the upper bound equal to one.

Introducing the inverse functions

$$\begin{aligned} g(z)&= \mathcal {F}_{1/2}^{-1}(z)\quad \text{ for } z\in (0,\infty ), \\ h(z)&= \mathcal {F}_{-1}^{-1}(z) = \log z - \log (1-z)\quad \text{ for } z\in (0,1), \end{aligned}$$

the transport equations can be written in a drift–diffusion form as:

1 $$\begin{aligned} \partial _t n - \operatorname {div}J_n = 0,&\quad J_n = n\nabla g(n) - n\nabla V, \end{aligned}$$

2 $$\begin{aligned} \partial _t p + \operatorname {div}J_p = 0,&\quad J_p = -(p\nabla g(p)+p\nabla V), \end{aligned}$$

3 $$\begin{aligned} \partial _t D + \operatorname {div}J_D = 0,&\quad J_D = -(D\nabla h(D)+D\nabla V) \quad \text{ in } \Omega ,\ t>0, \end{aligned}$$

where $\Omega \subset {{\mathbb {R}}}^d$ ($d\ge 1$) is a bounded domain. The electric potential is selfconsistently coupled to the charge densities by the Poisson equation:

4 $$\begin{aligned} \lambda ^2\Delta V = n-p-D+A(x)\quad \text{ in } \Omega , \end{aligned}$$

where $\lambda >0$ is the (scaled) Debye length and *A*(*x*) is the given dopant acceptor density. Following Strukov et al. (2009), we neglect recombination–generation effects. Equations (1)–(4) are supplemented with the initial and mixed Dirichlet–Neumann boundary conditions

5 $$\begin{aligned}&n(0,\cdot ) = n^I, \quad p(0,\cdot ) = p^I, \quad D(0,\cdot ) = D^I \quad \text{ in } \Omega , \end{aligned}$$

6 $$\begin{aligned}&n = \bar{n}, \quad p=\bar{p}, \quad V=\bar{V} \quad \text{ on } \Gamma _D,\ t>0, \end{aligned}$$

7 $$\begin{aligned}&J_n\cdot \nu = J_p\cdot \nu = \nabla V\cdot \nu = 0 \quad \text{ on } \Gamma _N,\ t>0, \end{aligned}$$

8 $$\begin{aligned}&J_D\cdot \nu = 0 \quad \text{ on } \partial \Omega , t>0. \end{aligned}$$

Here, $\Gamma _D$ is the union of Ohmic contacts and $\Gamma _N$ models the insulating boundary parts. Since the oxide vacancies are supposed not to leave the domain, we impose no-flux boundary conditions for *D* on the whole boundary. These boundary conditions are usually used in the literature (Greenlee et al. 2013; Strukov et al. 2009).

Although we consider general Fermi–Dirac statistics, our model contains some simplifications. First, we consider constant mobilities to simplify the calculations. A more general definition reads as, for instance, $J_n=m_n n\nabla \mu _n$ with the space-dependent mobility $m_n$. Second, we neglected recombination–generation terms. They can be included in the analysis, but we preferred to omit them for a clearer presentation. Third, we have chosen a constant semiconductor permittivity. It is possible to assume a space-dependent permittivity as long as it is strictly positive and bounded. For the two-species drift–diffusion model, the general physical situation was analyzed in Disser and Rehberg (2019).

The aim of this paper is to prove (i) the existence of global weak solutions (*n*, *p*, *D*, *V*) to (1)–(8) and (ii) the regularity $n,p,D\in L^\infty (0,T;L^\infty (\Omega ))$ for any $T>0$.

### Mathematical Difficulties

The misfit of the boundary conditions for (*n*, *p*) on the one hand and for *D* on the other hand gives the first main mathematical difficulty. A second difficulty comes from the fact that we consider three species instead of two charge carriers as done in many papers (Gajewski and Gröger 1989; Glitzky and Liero 2019; Jüngel 1996). Indeed, the two-species case allows one to exploit a monotonicity property of the drift term such that the quadratic nonlinearity can be handled (Gajewski and Gröger 1989). For more than two species, one may use Gagliardo–Nirenberg estimates, but this is possible in two space dimensions only (Glitzky and Hünlich 2005). This issue can be overcome by $W^{1,r}_\textrm{loc}(\Omega )$ estimates with $r>1$ (Jourdana et al. 2023), but leading to very weak solutions and boundedness of solutions in two space dimensions only. The third difficulty are the nonlinearities from the Fermi–Dirac statistics, which complicates the estimates. We prove in Appendix A that

9 $$\begin{aligned} g'(z) \sim z^{-1}\textrm{1}_{\{z\le \mathcal {F}_{1/2}(0)\}} + z^{-1/3}\textrm{1}_{\{z>\mathcal {F}_{1/2}(0)\}}\quad \text{ for } z>0, \end{aligned}$$

where $A\sim B$ means that there exist constants $C_1,C_2>0$ such that $C_1A\le B\le C_2A$. In particular, the nonlinear diffusion $n\nabla g(n)=ng'(n)\nabla n$ can be approximated by $\nabla n$ in the low-density regime and by $(3/5)\nabla n^{5/3}$ in the high-density regime. On the other hand, the Blakemore statistics gives to the diffusion $D\nabla h(D)=-\nabla \log (1-D)$, which exhibits a singularity at $D=1$. The technical issues associated with this singularity are overcome by using some ideas from Cancès et al. (2021), developed for a one-species model.

### State of the Art and Key Ideas

There are only a few works dealing with the drift–diffusion equations for more than two species. General existence results for an *n*-species model have been proved in Heibig et al. (2019) for an abstract drift operator satisfying smoothing conditions. In Choi and Lui (1995), Glitzky and Hünlich (1997, 2005) and Glitzky and Liero (2019), the existence of global weak solutions was shown in at most two space dimensions. The three-dimensional case was investigated in Bothe et al. (2014) using Robin boundary conditions for the electric potential. In the work (Bhattacharya et al. 2022), the function $n\nabla g(n)=\nabla (n+\eta n^q)$ with $\eta >0$ and $q\ge 4$ was chosen to regularize the diffusion term, which allows for an analysis in three space dimensions. The paper (Gajewski and Gröger 1996) studies the drift–diffusion equations with Fermi–Dirac statistics but assuming inhomogeneous Neumann boundary conditions on $\partial \Omega$. A drift–diffusion system with Fermi–Dirac statistics for electrons and holes and with Blakemore statistics for the ionic vacancy carriers, modeling perovskite solar cells, was analyzed recently in Abdel et al. (2025) in two space dimensions. A free energy inequality for this model in three space dimensions was shown in Abdel et al. (2023).

Our analysis is based, as in Abdel et al. (2023) and Gajewski and Gröger (1996), on estimates derived from the free energy inequality. The asymptotic behavior of the Fermi–Dirac integral $\mathcal {F}_{1/2}$ allows for an argument similar to Bhattacharya et al. (2022) but based on physical bounds. Indeed, the behavior (9) shows that the diffusion is given by:

$$\begin{aligned} n\nabla g(n) \sim n(n^{-1}+n^{-1/3})\nabla n = \nabla \bigg (n + \frac{3}{5} n^{5/3}\bigg ). \end{aligned}$$

The first term corresponds to linear diffusion, while the second term allows for higher integrability estimates. As a by-product, we are able to weaken the condition $q\ge 4$ in Bhattacharya et al. (2022) to $q\ge 5/3$. (By Jüngel and Vetter 2025, one may weaken this condition even to $q>6/5$.)

To specify the free energy inequality, we introduce the anti-derivatives of *g* and *h*,

10 $$\begin{aligned} G(s) = \int _{\mathcal {F}_{1/2}(0)}^s g(z)\textrm{d}z, \quad H(s) = \int _{\mathcal {F}_{-1}(0)}^s h(z)\textrm{d}z, \end{aligned}$$

the relative energy density

$$\begin{aligned} \mathcal {G}(s|\bar{s}) = G(s) - G(\bar{s}) - G'(\bar{s})(s-\bar{s}), \quad s,\bar{s}\ge 0, \end{aligned}$$

and the free energy

$$\begin{aligned} E(n,p,D,V) = \int _\Omega \bigg (\mathcal {G}(n|\bar{n}) + \mathcal {G}(p|\bar{p}) + H(D) + D\bar{V} + \frac{\lambda ^2}{2}|\nabla (V-\bar{V})|^2\bigg )\textrm{d}x. \end{aligned}$$

A formal computation, made rigorous in Theorem 1, shows that

11 $$\begin{aligned}&\frac{dE}{dt}(n,p,D,V) + \frac{1}{2}\int _\Omega \big ( n|\nabla (g(n)-V)|^2 + p|\nabla (g(p)+V)|^2 \nonumber \\&\quad + D|\nabla (h(D)+V)|^2\big )\textrm{d}x \le C(\bar{n},\bar{p},\bar{D},T). \end{aligned}$$

This yields a priori estimates for *n*, *p* in $L^\infty (0,T;L^{5/3}(\Omega ))$ and for *V* in $L^\infty (0,T;H^1(\Omega ))$. Moreover, defining $\widetilde{g}$ by $\widetilde{g}'(n)=\sqrt{n}g'(n)$,

$$\begin{aligned} |\nabla \widetilde{g}(n)| \le |\nabla \widetilde{g}(n)-\sqrt{n}\nabla V| + \sqrt{n}|\nabla V| = \sqrt{n}|\nabla (g(n)-V)| + \sqrt{n}|\nabla V| \end{aligned}$$

is uniformly bounded in $L^2(0,T;L^{5/4}(\Omega ))$. Unfortunately, this regularity is *not* sufficient to define $n\nabla g(n) = \sqrt{n}\nabla \widetilde{g}(n)$ since $\sqrt{n}$ is bounded in $L^\infty (0,T;L^{10/3}(\Omega ))$ and $3/10+4/5>1$. However, we are able to improve the regularity by an iteration argument to $\nabla \widetilde{g}(n)\in L^2(0,T;L^r(\Omega ))$ with $r<8/5$ (see Lemma 11), which is sufficient since $3/10+5/8<1$.

The treatment of the diffusion $D\nabla h(D)=-\nabla \log (1-D)$ is quite delicate because of the singularity at $D=1$. The idea is to approximate $L(D)=-\log (1-D)$ by regular functions $L_k$ with $k\in {{\mathbb {N}}}$. The identification of the limit of the sequence $L_k(D_k)$ of approximating solutions $D_k$, which converge strongly to some function *D*, is then achieved by a monotonicity argument (Minty trick); see Lemma 16. These ideas allow us to prove the existence of global weak solutions.

The second main result is the boundedness of weak solutions. The difficulty comes from the estimate of the quadratic drift terms, which can be overcome in the case of two species by a monotonicity argument. For more than two species, we use the Gagliardo–Nirenberg inequality to estimate this term, similarly as in Glitzky and Hünlich (1997) for two space dimensions. In three dimensions, we need as in Jüngel and Vetter (2025) the elliptic regularity result $V\in W^{1,r}(\Omega )$ with $r>3$. This is possible even under mixed boundary conditions if $\Gamma _D$ and $\Gamma _N$ do not meet in a “too wild” manner (Disser and Rehberg 2015, Theorem 4.8). Then, applying an Alikakos-type iteration argument similar to Jourdana et al. (2023), Jüngel and Vetter (2025), we obtain *q*-uniform estimates in $L^\infty (0,T;L^{q}(\Omega ))$ for any $q<\infty$. The boundedness follows after performing the limit $q\rightarrow \infty$.

### Main Results

First, we introduce some notation. We set $\Omega _T=\Omega \times (0,T)$ for $T>0$, denote by $\textrm{m}(B)$ the measure of a set $B\subset {{\mathbb {R}}}^d$, and set for $1\le q\le \infty$,

$$\begin{aligned} W_D^{1,q}(\Omega ) = \{u\in W^{1,q}(\Omega ): u=0 \text{ on } \Gamma _D\}, \quad H_D^1(\Omega ) = W_D^{1,2}(\Omega ). \end{aligned}$$

The function $V^I\in H_D^1(\Omega )+\bar{V}$ is the unique solution to

$$\begin{aligned} \lambda ^2\Delta V^I = n^I-p^I-D^I+A(x) \text{ in } \Omega , \quad V^I=\bar{V} \text{ on } \Gamma _D, \quad \nabla V^I\cdot \nu = 0 \text{ on } \Gamma _N. \end{aligned}$$

Constants $C>0$ are generic and may change their value from line to line.

We impose the following assumptions.

1. Domain: $\Omega \subset {{\mathbb {R}}}^d$ $(1\le d\le 3)$ is a bounded domain with Lipschitz boundary, $\partial \Omega = \Gamma _D \cup \Gamma _N$, $\Gamma _D \cap \Gamma _N = \emptyset$, $\textrm{m}(\Gamma _D) > 0$, and $\Gamma _N$ is relatively open in $\partial \Omega$.
2. Data: $$T > 0$$, $$\lambda > 0$$, $$A \in L^\infty (\Omega )$$.
3. Boundary data: $\bar{n}$, $\bar{p}$, $\bar{V} \in W^{1,\infty }(\Omega )$ with $\bar{n}$, $\bar{p} > 0$ in $\Omega$.
4. Initial data: $n^I$, $p^I$, $D^I \in L^2(\Omega )$ satisfy $n^I$, $p^I$, $D^I \ge 0$ in $\Omega$, $E(n^I, p^I, D^I, V^I)$ $< \infty$. Furthermore, $\sup _\Omega D^I\le 1$ and
  $$\begin{aligned} D_\Omega ^I:=\frac{1}{\textrm{m}(\Omega )}\int _\Omega D^I \textrm{d}x < 1. \end{aligned}$$
5. Elliptic Regularity: There exists $r>3$ such that for some constant $C > 0$ and all $f \in L^{3r/(r+3)}(\Omega )$ the weak solution *V* to the Poisson problem
  12 $$\begin{aligned} \Delta V = f \text{ in } \Omega , \quad V = \bar{V} \text{ on } \Gamma _D, \quad \nabla V \cdot \nu = 0 \text{ on } \Gamma _N, \end{aligned}$$
  satisfies the estimate
  13 $$\begin{aligned} \Vert V \Vert _{W^{1,r}(\Omega )} \le C \Vert f\Vert _{L^{3r/(r+3)}(\Omega )} + C. \end{aligned}$$

Let us discuss the assumptions. We can assume higher space dimensions in most of the estimates, but we restrict ourselves to $d\le 3$ because of the applications. The boundary data in Assumption (A3) is assumed to be independent of time to simplify the computations; time-dependent boundary data are possible, see, e.g., Degond et al. (1997, Sec. 2). Compared to Abdel et al. (2025), we do not need pointwise positive lower bounds of the densities and we can allow for vacuum as well as saturation of the oxygen vacancy density. We only prevent $D_\Omega ^I=1$ in Assumption (A4), which would be physically unrealistic.

The most restrictive condition is Assumption (A5). Indeed, we can only expect the regularity $V\in W^{1,r}(\Omega )$ with $r>2$ for the solution *V* to (12) with mixed boundary conditions (Gröger 1994). The regularity $r>3$ can be achieved under reasonable conditions on $\Gamma _D$ and $\Gamma _N$ (Disser and Rehberg 2015, Theorem 4.8). These conditions are satisfied if $\Gamma _D$ and $\Gamma _N$ intersect with an “angle” not larger than $\pi$ (Disser and Rehberg 2015, Prop. 3.4). Assumption (A5) is *not* needed for the existence result but for the proof of the boundedness of solutions.

Our first main result is the existence of global weak solutions.

#### Theorem 1

(Global existence) Let Assumptions (A1)–(A4) hold. Then, there exists a weak solution (*n*, *p*, *D*, *V*) to (1)–(8) satisfying $n,p\ge 0$, $0\le D<1$ a.e. in $\Omega _T$,

$$\begin{aligned}&n,p\in L^\infty (0,T;L^{5/3}(\Omega )) \cap L^2(0,T;W^{1,\alpha }(\Omega )), \quad D\in L^\infty (\Omega _T)\cap L^2(0,T;H^1(\Omega )), \\&n\nabla g(n),p\nabla g(p)\in L^2(0,T;L^{5/4}(\Omega )), \quad D\nabla h(D)\in L^2(0,T;L^{2}(\Omega )), \\&\partial _t n,\partial _t p\in L^{7/5}(0,T;W_D^{1,2\alpha /(4-\alpha )}(\Omega )), \quad \partial _t D\in L^2(0,T;H^1(\Omega )'), \quad V\in L^\infty (0,T;H^1(\Omega )), \end{aligned}$$

where $\alpha <8/5$ if $d=3$, $\alpha <2$ if $d=2$, and $\alpha =2$ if $d=1$. The fluxes are understood in the sense

$$\begin{aligned} J_n&= n\nabla (g(n)-V)\in L^2(0,T;L^{5/4}(\Omega )), \\ J_p&= -p\nabla (g(p)+pV)\in L^2(0,T;L^{5/4}(\Omega )), \\ J_D&= -D\nabla (h(D)+V) = -\nabla \log (1-D)+D\nabla V\in L^2(\Omega _T). \end{aligned}$$

The solution satisfies the free energy inequality

14 $$\begin{aligned}&E(n,p,D,V)(t) + \frac{1}{2}\int _0^t\int _\Omega \big ( n|\nabla (g(n)-V)|^2 + p|\nabla (g(p)+V)|^2 \nonumber \\&\quad + D|\nabla (h(D)+V)|^2\big )\textrm{d}x\textrm{d}s \le C(E^I,\Lambda ,T), \end{aligned}$$

where $$E^I:=E(n^I,p^I,D^I,V^I)$$,

$$\begin{aligned} \Lambda := 2\big (\Vert \nabla (g(\bar{n})-\bar{V})\Vert _{L^\infty (\Omega )}^2 + \Vert \nabla (g(\bar{p})+\bar{V})\Vert _{L^\infty (\Omega )}^2\big ), \end{aligned}$$

and it holds that $C(E^I,\Lambda ,T)=0$ if $\Lambda =0$.

The property $\Lambda =0$ means that the boundary data is in thermal equilibrium. In this situation, the free energy is a Lyapunov functional. For the proof of Theorem 1, we first approximate the problem by truncating the nonlinearities (densities) in the diffusion and drift terms and prove the existence of approximate solutions by using the Leray–Schauder fixed-point theorem. The compactness of the fixed-point operator is a consequence of the approximate free energy inequality. From this inequality, we derive uniform bounds for the approximate solutions, allowing us to take the re-regularizing limit. As mentioned before, the main difficulties are the derivation of improved estimates via an iteration argument and the treatment of the singularity $D=1$.

Our second main result is the boundedness of weak solutions.

#### Theorem 2

(Boundedness) Let Assumptions (A1)–(A5) hold and assume that $n^I$, $p^I$, $D^I\in L^\infty (\Omega )$. Then, the weak solution constructed in Theorem 1 satisfies

$$\begin{aligned} n,p,D\in L^\infty (\Omega _T), \quad V\in L^{\infty }(0,T;W^{1,r}(\Omega )) \subset L^\infty (\Omega _T), \end{aligned}$$

where $r>3$ is given in Assumption (A5).

The restriction to three space dimensions comes from regularity (13). The boundedness result is not surprising in view of Jüngel and Vetter (2025, Theorem 2). Indeed, since $ng'(n)\sim 1+n^{2/3}$, the diffusion term contains the porous-medium term $\nabla n^{5/3}$, and it is proved in Jüngel and Vetter (2025) that this nonlinear diffusion leads to an improvement of the integrability of the densities up to $L^\infty (\Omega )$. The idea is first to prove that $n,p\in L^\infty (0,T;L^2(\Omega ))$. This is used as the starting point of a recursion, showing that $n,p\in L^\infty (0,T;L^q(\Omega ))$ for any $q<\infty$, but with bounds that may depend on *q*. This allows us to use $n^q-\bar{n}^q$, $p^q-\bar{p}^q$ as test functions in the weak formulations to (1), (2), respectively. By an Alikakos iteration, it turns out that the $L^\infty (0,T;L^q(\Omega ))$ bounds are independent of *q*, and we can pass to the limit $q\rightarrow \infty$ to conclude. To reduce the technicalities and since the first parts of the proof are technically similar to Jüngel and Vetter (2025, Sec. 3), we detail only the last part of the proof (the Alikakos argument).

#### Remark 3

(Generalization) Our results hold for an arbitrary number of charged particles, since we use the Poisson equation only through the norm estimates for *V* and $\nabla V$. In particular, we can consider the transport equations

$$\begin{aligned} \partial _t u_i&= \operatorname {div}(u_i\nabla g(u_i) + z_iu_i\nabla V), \quad i\in I, \\ \partial _t u_i&= \operatorname {div}(u_i\nabla h(u_i) + z_iu_i\nabla V), \quad i\in I_0, \\ \lambda ^2\Delta V&= -\sum _{i\in I\cup I_0}z_iu_i + A(x)\quad \text{ in } \Omega ,\ t>0, \end{aligned}$$

where $z_i\in {{\mathbb {R}}}$ are the particle charges, $I,I_0\subset {{\mathbb {N}}}$ are some index sets, and the initial and boundary conditions are as in (5)–(8). $\square$

The paper is organized as follows: Theorems 1 and 2 are proved in Sects. 2 and 3, respectively. Auxiliary inequalities involving Fermi–Dirac integrals are proved in Appendix A. We also need a nonlinear version of the Poincaré–Wirtinger inequality, which is shown in Appendix B.

## Proof of Theorem 1

We prove the existence of global weak solutions to (1)–(8). To this end, we truncate the coefficients in the parabolic equations with parameter $k\in {{\mathbb {N}}}$, solve the corresponding approximate problem, derive uniform estimates from an approximate free energy inequality, and pass to the limit $k\rightarrow \infty$.

### Approximate Problem

We introduce for $k\in {{\mathbb {N}}}$ and $z\in {{\mathbb {R}}}$ the truncations $T_k(z)=\max \{0,\min \{k,z\}\}$ and

$$\begin{aligned} S_k^1(z) = {\left\{ \begin{array}{ll} 1 & \text{ for } z\le 0, \\ zg'(z) & \text{ for } 0<z\le k, \\ k^{2/3}z^{1/3}g'(z) & \text{ for } z>k, \end{array}\right. } \quad S_k^2(z) = {\left\{ \begin{array}{ll} 1 & \text{ for } z\le 0, \\ zh'(z) & \text{ for } 0<z\le k/(k+1), \\ 1+k & \text{ for } z>k/(k+1). \end{array}\right. } \end{aligned}$$

The functions $S_k^1$ and $S_k^2$ are continuous, bounded, and strictly positive on ${{\mathbb {R}}}$, noting that $zh'(z)=1/(1-z)$ for $z\in (0,1)$. The approximate problem reads as follows:

15 $$\begin{aligned} \partial _t n_k&= \operatorname {div}\big (S_k^1(n_k)\nabla n_k - T_k(n_k)\nabla V_k\big ), \end{aligned}$$

16 $$\begin{aligned} \partial _t p_k&= \operatorname {div}\big (S_k^1(p_k)\nabla p_k + T_k(p_k)\nabla V_k\big ), \end{aligned}$$

17 $$\begin{aligned} \partial _t D_k&= \operatorname {div}\big (S_k^2(D_k)\nabla D_k + T_{k/(k+1)}(D_k)\nabla V_k \big ), \end{aligned}$$

18 $$\begin{aligned} \lambda ^2\Delta V_k&= n_k-p_k-D_k+A(x)\quad \text{ in } \Omega ,\ t>0, \end{aligned}$$

with the initial and boundary conditions (5)–(8), where (*n*, *p*, *D*, *V*) is replaced by $$(n_k,p_k,$$ $$D_k,V_k)$$. Clearly, if $$k\rightarrow \infty $$, we recover formulation (1)–(3). The truncation $$T_{k/(k+1)}(D_k)$$ is chosen since we expect that the limit *D* of $$D_k$$ satisfies $$D<1$$ a.e.

We show the existence of solutions to (15)–(18) by using a fixed-point argument. For this, let $(n^*,p^*,D^*)\in L^2(\Omega _T)^3$ and $\sigma \in [0,1]$. We apply (Zeidler 1990, Theorem 23.A) to infer that the linearized problem

19 $$\begin{aligned} \partial _t n&= \operatorname {div}\big (S_k^1(n^*)\nabla n - \sigma T_k(n^*)\nabla V\big ), \end{aligned}$$

20 $$\begin{aligned} \partial _t p&= \operatorname {div}\big (S_k^1(p^*)\nabla p + \sigma T_k(p^*)\nabla V\big ), \end{aligned}$$

21 $$\begin{aligned} \partial _t D&= \operatorname {div}\big (S_k^2(D^*)\nabla D + \sigma T_{k/(k+1)}(D^*)\nabla V \big ), \end{aligned}$$

22 $$\begin{aligned} \lambda ^2\Delta V&= n^*-p^*-D^*+\sigma A(x) \quad \text{ in } \Omega ,\ t>0, \end{aligned}$$

with the initial and boundary conditions

$$\begin{aligned}&n(0,\cdot ) = \sigma n^I, \quad p(0,\cdot ) = \sigma p^I, \quad D(0,\cdot ) = \sigma D^I \quad \text{ in } \Omega , \\&n = \sigma \bar{n}, \quad p=\sigma \bar{p}, \quad V=\sigma \bar{V} \quad \text{ on } \Gamma _D,\ t>0, \\&\nabla n\cdot \nu = \nabla p\cdot \nu = \nabla V\cdot \nu = 0 \quad \text{ on } \Gamma _N,\ t>0, \\&\big (S_k^2(D^*)\nabla D + \sigma T_{k/(k+1)}(D^*)\nabla V \big )\cdot \nu = 0 \quad \text{ on } \partial \Omega , t>0, \end{aligned}$$

has a unique solution $(n,p,D,V)\in L^2(0,T;H^1(\Omega ))^4$ such that $n,p,D\in H^1(0,T;H^1_D(\Omega )')$. This defines the fixed-point operator $F:L^2({\Omega _T})^3\times [0,1]\rightarrow L^2(\Omega _T)^3$, $(n^*,p^*,D^*;\sigma )\mapsto (n,p,D)$. Standard arguments show that *F* is continuous and satisfies $F(n^*,p^*,D^*;0)=(0,0,0)$. To apply the Leray–Schauder fixed-point theorem, we need to find a uniform bound for all fixed points of $F(\cdot ,\cdot ,\cdot ;\sigma )$.

#### Lemma 4

Let (*n*, *p*, *D*) be a fixed point of $F(\cdot ,\cdot ,\cdot ;\sigma )$, where $\sigma \in [0,1]$. Then (*n*, *p*, *D*) is bounded in $L^\infty (0,T;L^2(\Omega ))\cap L^2(0,T;H^1(\Omega ))$ uniformly in $\sigma$.

#### Proof

Since the proof is similar to that one of Jourdana et al. (2023, Lemma 2.1), we only sketch it. Let $(n^*,p^*,D^*)=(n,p,D)$ be a fixed point of $F(\cdot ,\cdot ,\cdot ;\sigma )$. We use the test function $V-\sigma \bar{V}$ in the weak formulation of (22) and apply the Young and Poincaré inequality to find that

$$\begin{aligned} \int _0^T\int _\Omega |\nabla V|^2 \textrm{d}x\textrm{d}t \le C + C\int _0^T\int _\Omega (n^2+p^2+D^2)\textrm{d}x\textrm{d}t, \end{aligned}$$

where $C>0$ is a constant independent of $(n,p,D,\sigma )$. Next, we use the test function $n-\sigma \bar{n}$ in the weak formulation of (19) and take into account that $S_k^1(n)\ge c(k)>0$ and $S_k^2(n)\ge 1$. Then, with the Young inequality,

$$\begin{aligned}&\frac{1}{2}\int _\Omega (n(t)-\sigma \bar{n})^2 \textrm{d}x - \frac{1}{2}\int _\Omega (n^I-\sigma \bar{n})^2 \textrm{d}x + \int _0^t\int _\Omega |\nabla n|^2 \textrm{d}x\textrm{d}s \\&\quad \le C + C(k)\int _0^t\int _\Omega |\nabla V|^2 \textrm{d}x\textrm{d}s \le C + C\int _0^t\int _\Omega (n^2+p^2+D^2)\textrm{d}x\textrm{d}s. \end{aligned}$$

We derive similar estimates when using $p-\sigma \bar{p}$ and *D* in the weak formulations of (20) and (21), respectively. Adding these estimates yields

$$\begin{aligned}&\int _\Omega \big (n(t)^2+p(t)^2+D(t)^2\big )\textrm{d}x + \int _0^t\int _\Omega \big (|\nabla n|^2+|\nabla p|^2+|\nabla D|^2\big )\textrm{d}x\textrm{d}s \\&\quad \le C + C\int _0^t\int _\Omega (n^2+p^2+D^2)\textrm{d}x\textrm{d}s. \end{aligned}$$

We deduce from Gronwall’s lemma $\sigma$-uniform bounds for (*n*, *p*, *D*)  in $L^\infty (0,T;L^2(\Omega ))\cap L^2(0,T;H^1(\Omega ))$. $\square$

The bounds in Lemma 4 imply uniform estimates for $(\partial _t n,\partial _t p,\partial _t D)$ in $L^2(0,T;H^1_D(\Omega )')$. By the Aubin–Lions lemma, the embedding $L^2(0,T;H^1(\Omega ))\cap H^1(0,T;H^1_D(\Omega )')\hookrightarrow L^2(\Omega _T)$ is compact. Thus, $F:L^2(\Omega _T)^3\times [0,1]\rightarrow L^2(\Omega _T)^3$ is compact. The assumptions of the Leray–Schauder fixed-point theorem are satisfied, and we conclude the existence of a fixed point of $F(\cdot ,\cdot ,\cdot ;1)$, i.e. a solution to (15)–(18) and (5)–(8). We summarize:

#### Lemma 5

(Existence for the approximate problem) Let Assumptions (A1)–(A4) hold. Then, there exists a weak solution to (15)–(18) with initial and boundary conditions (5)–(8).

The solution $(n_k,p_k,D_k)$ to (15)–(18) is componentwise nonnegative. Indeed, using the test function $n_k^-=\min \{0,n_k\}$ in the weak formulation of (15), we have

$$\begin{aligned} \frac{1}{2}\int _\Omega (n_k^-)(t)^2 \textrm{d}x + \int _0^t\int _\Omega S_k^1(n_k)|\nabla n_k^-|^2 \textrm{d}x\textrm{d}s = \int _0^t\int _{\{n_k<0\}} T_k(n_k)\nabla V_k\cdot \nabla n_k \textrm{d}x\textrm{d}s = 0, \end{aligned}$$

since $T_k(n_k)=0$ for $n_k<0$, showing that $n_k^-(t)=0$ and consequently $n_k(t)\ge 0$ for $t>0$. We note that the mass of the oxide vacancies is conserved,

$$\begin{aligned} \int _\Omega D_k(t)\textrm{d}x = \int _\Omega D^I \textrm{d}x\quad \text{ for } t>0, \end{aligned}$$

while this is generally not the case for the electron and hole densities because of the Dirichlet boundary conditions.

### Approximate Energy Inequality

We derive the approximate analog of the free energy inequality (11). Similarly as in Jourdana et al. (2023, Sec. 2.3), we define for $0<\delta <\mathcal {F}_{1/2}(0)$ the approximations

23 $$\begin{aligned} \begin{aligned} G_{k,\delta }(s)&= \int _{\mathcal {F}_{1/2}(0)}^s\int _{\mathcal {F}_{1/2}(0)}^y \frac{S_k^1(z)}{T_k(z)+\delta }\textrm{d}z\textrm{d}y, \quad \widetilde{g}_{k,\delta }(s) = \int _0^s \frac{S_k^1(y)}{\sqrt{T_k(y)+\delta }}\textrm{d}y, \\ H_{k,\delta }(s)&= \int _{\mathcal {F}_{-1}(0)}^s\int _{\mathcal {F}_{-1}(0)}^y \frac{S_k^2(z)}{T_{k/(k+1)}(z)+\delta }\textrm{d}z\textrm{d}y, \quad \widetilde{h}_{k,\delta }(s) = \int _0^s \frac{S_k^2(y)}{\sqrt{T_{k/(k+1)}(y)+\delta }}\textrm{d}y. \end{aligned} \end{aligned}$$

Recalling that $S_k^1(z)\rightarrow zg'(z)$, $S_k^2(z)\rightarrow zh'(z)$, and $T_k(z)\rightarrow z$ pointwise as $k\rightarrow \infty$, the functions $G_{k,\delta }$ and $H_{k,\delta }$ approximate the anti-derivatives of *g* and *h*, respectively (see (10)), while $\widetilde{g}_{k,\delta }$ and $\widetilde{h}_{k,\delta }$ approximate

$$\begin{aligned} \widetilde{g}(s)&:= \int _{\mathcal {F}_{1/2}(0)}^{s}\sqrt{z}g'(z)\textrm{d}z, \\ \widetilde{h}(s)&:= \int _{\mathcal {F}_{-1}(0)}^s\sqrt{z}h'(z)\textrm{d}z = 2\tanh ^{-1}(\sqrt{s}) - 2\tanh ^{-1}(1/\sqrt{2}), \end{aligned}$$

respectively, since $\mathcal {F}_{-1}(0)=1/2$. These definitions yield the following chain rules:

24 $$\begin{aligned} \begin{aligned} S_k^1(n_k)\nabla n_k&= \sqrt{T_k(n_k)+\delta } \nabla \widetilde{g}_{k,\delta }(n_k), \\ S_k^2(D_k)\nabla D_k&= \sqrt{T_{k/(k+1)}(D_k)+\delta } \nabla \widetilde{h}_{k,\delta }(D_k), \end{aligned} \end{aligned}$$

and similarly for $p_k$ instead of $n_k$. They are the truncated analogs of the chain rules $n_k g'(n_k)\nabla n_k=\sqrt{n_k}\nabla \widetilde{g}(n_k)$ and $D_k h'(n_k)\nabla D_k=\sqrt{D_k}\nabla \widetilde{h}(D_k)$. Choosing $\delta =0$ in (24), we see that the approximate fluxes can be formulated as:

25 $$\begin{aligned} \begin{aligned} S_k^1(n_k)\nabla n_k - T_k(n_k)\nabla V_k&= \sqrt{T_k(n_k)}\big (\nabla \widetilde{g}_k(n_k) - \sqrt{T_k(n_k)}\nabla V_k\big ), \\ S_k^1(p_k)\nabla p_k + T_k(p_k)\nabla V_k&= \sqrt{T_k(p_k)}\big (\nabla \widetilde{g}_k(p_k) + \sqrt{T_k(p_k)}\nabla V_k\big ), \\ S_k^2(D_k)\nabla D_k + T_k(D_k)\nabla V_k&= \sqrt{T_k(D_k)}\big (\nabla \widetilde{h}_k(D_k) + \sqrt{T_k(D_k)}\nabla V_k\big ). \end{aligned} \end{aligned}$$

Next, we define for $s,\bar{s}\ge 0$ the approximate relative energies

26 $$\begin{aligned} \mathcal {G}_{k,\delta }(s|\bar{s}) = G_{k,\delta }(s) - G_{k,\delta }(\bar{s}) - G_{k,\delta }'(\bar{s})(s-\bar{s}), \quad \mathcal {H}_{k,\delta }(s) = H_{k,\delta }(s) + s\bar{V} \end{aligned}$$

and the approximate free energy

27 $$\begin{aligned} E_{k,\delta }(n_k,p_k,D_k,V_k) = \int _\Omega \bigg ( \mathcal {G}_{k,\delta }(n_k|\bar{n}) + \mathcal {G}_{k,\delta }(p_k|\bar{p}) + \mathcal {H}_{k,\delta }(D_k) + \frac{\lambda ^2}{2}|\nabla (V-\bar{V})|^2\bigg )\textrm{d}x. \end{aligned}$$

We set

28 $$\begin{aligned} E_{k,\delta }^I&:= E_{k,\delta }(n^I,p^I,D^I,V^I), \end{aligned}$$

29 $$\begin{aligned} \Lambda _{k,\delta }&:= 2\Vert \nabla (G'_{k,\delta }(\bar{n})-\bar{V}) \Vert _{L^\infty (\Omega )}^2 + 2\Vert \nabla (G'_{k,\delta }(\bar{p})+\bar{V}) \Vert _{L^\infty (\Omega )}^2. \end{aligned}$$

For the derivation of the approximate free energy inequality, we need the following lemma.

#### Lemma 6

There exists a constant $C>0$ such that for any $k,\delta >0$ satisfying $0<\delta<\mathcal {F}_{1/2}(0)<k$,

$$\begin{aligned} T_k(s)^{5/3} \le C(1+G_{k,\delta }(s)) \quad \text{ for } s>0. \end{aligned}$$

#### Proof

Let $0<s\le \mathcal {F}_{1/2}(0)$. Since $s<k$, we have $T_k(s)^{5/3}=s^{5/3}\le \mathcal {F}_{1/2}(0)^{5/3}\le C$. Next, let $\mathcal {F}_{1/2}(0)<s\le k$. Then, $S_k^1(s)=sg(s)$ and, by Lemma 20,

$$\begin{aligned} G_{k,\delta }(s)&= \int _{\mathcal {F}_{1/2}(0)}^s\int _{\mathcal {F}_{1/2}(0)}^y \frac{zg'(z)}{z+\delta }\textrm{d}z\textrm{d}y \ge C\int _{\mathcal {F}_{1/2}(0)}^s\int _{\mathcal {F}_{1/2}(0)}^y \frac{z(z^{-1}+z^{-1/3})}{z+\delta }\textrm{d}z\textrm{d}y \\&\ge \frac{C}{2}\int _{\mathcal {F}_{1/2}(0)}^s\int _{\mathcal {F}_{1/2}(0)}^y z^{-1/3}\textrm{d}z\textrm{d}y, \end{aligned}$$

since $\delta <\mathcal {F}_{1/2}(0)\le z$. An integration of the right-hand side leads to

$$\begin{aligned} G_{k,\delta }(s) \ge \frac{3C}{4}\bigg (\frac{3}{5} s^{5/3} - \mathcal {F}_{1/2}(0)^{2/3}s + \frac{2}{5}\mathcal {F}_{1/2}(0)^{5/3}\bigg ) \ge CT_k(s)^{5/3} - C. \end{aligned}$$

Finally, if $s>k$, we have $T_k(s)^{5/3}=k^{5/3}\le CG_{k,\delta }(k)\le CG_{k,\delta }(s)$. This finishes the proof. $\square$

#### Lemma 7

(Approximate free energy inequality for $E_{k,\delta }$) Let Assumptions (A1)–(A4) hold and let $(n_k,p_k,D_k,V_k)$ be the weak solution constructed in Lemma 5. Then, for all $0<t<T$,

30 $$\begin{aligned}&E_{k,\delta }(n_k,p_k,D_k,V_k)(t) + \frac{1}{2}\int _0^t\int _\Omega \big |\nabla \widetilde{g}_{k,\delta }(n_k) - \sqrt{T_k(n_k)+\delta }\nabla V_k\big |^2 \textrm{d}x\textrm{d}s \nonumber \\&\qquad + \frac{1}{2}\int _0^t\int _\Omega \big |\nabla \widetilde{g}_{k,\delta }(p_k) + \sqrt{T_k(p_k)+\delta }\nabla V_k\big |^2 \textrm{d}x\textrm{d}s \nonumber \\&\qquad + \frac{1}{2}\int _0^t\int _\Omega \big |\nabla \widetilde{h}_{k,\delta }(D_k) + (T_{k/(k+1)}(D_k)+\delta )^{1/2}\nabla V_k\big |^2 \textrm{d}x\textrm{d}s \nonumber \\&\quad \le C(E_{k,\delta }^I,\Lambda _{k,\delta },T). \end{aligned}$$

The constant $C(E_{k,\delta }^I,\Lambda _{k,\delta },T)\ge 0$ vanishes if $\Lambda _{k,\delta }=0$ and $\delta =0$.

#### Proof

We use the test function $G'_{k,\delta }(n_k)-G'_{k,\delta }(\bar{n}) - V_k + \bar{V}$ (see definition (23)) in the weak formulation of (15):

$$\begin{aligned}&\big \langle \partial _t n_k, G'_{k,\delta }(n_k)-G'_{k,\delta }(\bar{n}) - V_k + \bar{V}\big \rangle \\&\quad = -\int _\Omega \big (S_k^1(n_k)\nabla n_k - T_k(n_k)\nabla V_k\big ) \cdot \nabla \big ((G'_{k,\delta }(n_k) - V_k) - (G'_{k,\delta }(\bar{n}) - \bar{V})\big )\textrm{d}x. \end{aligned}$$

We use the identities

$$\begin{aligned} \big \langle \partial _t n_k, G'_{k,\delta }(n_k)-G'_{k,\delta }(\bar{n}) \big \rangle&= \frac{\textrm{d}}{\textrm{d}t}\int _\Omega \big (G_{k,\delta }(n_k) - G_{k,\delta }(\bar{n}) - G'_{k,\delta }(\bar{n})(n_k-\bar{n})\big )\textrm{d}x, \\ S_k^1(n_k)\nabla n_k - T_k(n_k)\nabla V_k&= \sqrt{T_k(n_k)+\delta }\big (\nabla \widetilde{g}_{k,\delta }(n_k) - \sqrt{T_k(n_k)+\delta }\nabla V_k\big ) + \delta \nabla V_k, \\ \nabla (G'_{k,\delta }(n_k)-V_k)&= \frac{\nabla \widetilde{g}_{k,\delta }(n_k) - \sqrt{T_k(n_k)+\delta }\nabla V_k}{\sqrt{T_k(n_k)+\delta }}, \end{aligned}$$

which follow from the chain rules (24), to obtain

31 $$\begin{aligned}&\frac{\textrm{d}}{\textrm{d}t}\int _\Omega \big (G_{k,\delta }(n_k) - G_{k,\delta }(\bar{n}) - G'_{k,\delta }(\bar{n})(n_k-\bar{n})\big )\textrm{d}x - \big \langle \partial _t n_k,V_k-\bar{V}\big \rangle \nonumber \\&\quad = -\int _\Omega \big |\nabla \widetilde{g}_{k,\delta }(n_k) - \sqrt{T_k(n_k)+\delta }\nabla V_k\big |^2 \textrm{d}x \nonumber \\&\qquad - \int _\Omega \frac{\delta }{\sqrt{T_k(n_k)+\delta }}\nabla V_k \cdot \big (\nabla \widetilde{g}_{k,\delta }(n_k) - \sqrt{T_k(n_k)+\delta }\nabla V_k\big )\textrm{d}x \nonumber \\&\qquad + \int _\Omega \sqrt{T_k(n_k)+\delta } \big (\nabla \widetilde{g}_{k,\delta }(n_k) - 
\sqrt{T_k(n_k)+\delta }\nabla V_k\big ) \cdot \nabla (G'_{k,\delta }(\bar{n})-\bar{V})\textrm{d}x \nonumber \\&\qquad + \delta \int _\Omega \nabla V_k\cdot \nabla (G'_{k,\delta }(\bar{n})-\bar{V})\textrm{d}x \nonumber \\&\quad \le -\frac{1}{2}\int _\Omega \big |\nabla \widetilde{g}_{k,\delta }(n_k) - \sqrt{T_k(n_k)+\delta }\nabla V_k\big |^2 \textrm{d}x + \frac{\delta }{2}\int _\Omega |\nabla (G'_{k,\delta }(\bar{n})-\bar{V})|^2 \textrm{d}x \nonumber \\&\qquad + \frac{3\delta }{2}\int _\Omega |\nabla V_k|^2 \textrm{d}x + \Vert \nabla (G'_{k,\delta }(\bar{n})-\bar{V})\Vert _{L^\infty (\Omega )}^2 \int _\Omega (T_k(n_k)+\delta )\textrm{d}x, \end{aligned}$$

where we used the inequality $\delta /(\sqrt{T_k(n_k)+\delta })\le \sqrt{\delta }$ and Young’s inequality in the last step. Similarly, the test function $G'_{k,\delta }(p_k)-G'_{k,\delta }(\bar{p}) + V_k - \bar{V}$ in the weak formulation of (16) leads to

$$\begin{aligned}&\frac{\textrm{d}}{\textrm{d}t}\int _\Omega \big (G_{k,\delta }(p_k) - G_{k,\delta }(\bar{p}) - G'_{k,\delta }(\bar{p})(p_k-\bar{p})\big )\textrm{d}x + \big \langle \partial _t p_k,V_k-\bar{V}\big \rangle \\&\quad \le -\frac{1}{2}\int _\Omega \big |\nabla \widetilde{g}_{k,\delta }(p_k) + \sqrt{T_k(p_k)+\delta }\nabla V_k\big |^2 \textrm{d}x + \frac{\delta }{2}\int _\Omega |\nabla (G'_{k,\delta }(\bar{p})+\bar{V})|^2 \textrm{d}x \\&\qquad + \frac{3\delta }{2}\int _\Omega |\nabla V_k|^2 \textrm{d}x + \Vert \nabla (G'_{k,\delta }(\bar{p})+\bar{V})\Vert _{L^\infty (\Omega )}^2 \int _\Omega (T_k(p_k)+\delta )\textrm{d}x. \end{aligned}$$

Similarly, with the test function $H'_{k,\delta }(D_k)+V_k$ in the weak formulation of (17),

32 $$\begin{aligned}&\frac{\textrm{d}}{\textrm{d}t}\int _\Omega \mathcal {H}_{k,\delta }(D_k)\textrm{d}x + \langle \partial _t D_k,V_k-\bar{V}\rangle = \langle \partial _t D_k,H'_{k,\delta }(D_k) + V_k\rangle \nonumber \\&\quad \le -\frac{1}{2}\int _\Omega \big |\nabla \widetilde{h}_{k,\delta }(D_k) + (T_{k/(k+1)}(D_k)+\delta )^{1/2}\nabla V_k\big |^2 \textrm{d}x + \frac{\delta }{2}\int _\Omega |\nabla V_k|^2 \textrm{d}x. \end{aligned}$$

We add (31)–(32) and take into account definition (26) of the relative energies and definition (29) of $\Lambda _{k,\delta }$:

33 $$\begin{aligned}&\frac{\textrm{d}}{\textrm{d}t}\int _\Omega \big (\mathcal {G}_{k,\delta }(n_k|\bar{n}) + \mathcal {G}_{k,\delta }(p_k|\bar{p}) + \mathcal {H}_{k,\delta }(D_k)\big )\textrm{d}x - \big \langle \partial _t(n_k-p_k-D_k),V_k-\bar{V}\big \rangle \nonumber \\&\qquad + \frac{1}{2}\int _\Omega \big |\nabla \widetilde{g}_{k,\delta }(n_k) - \sqrt{T_k(n_k)+\delta }\nabla V_k\big |^2 \textrm{d}x \nonumber \\&\qquad + \frac{1}{2}\int _\Omega \big |\nabla \widetilde{g}_{k,\delta }(p_k) + \sqrt{T_k(p_k)+\delta }\nabla V_k\big |^2 \textrm{d}x \nonumber \\&\qquad + \frac{1}{2}\int _\Omega \big |\nabla \widetilde{h}_{k,\delta }(D_k) + (T_{k/(k+1)}(D_k)+\delta )^{1/2}\nabla V_k\big |^2 \textrm{d}x \nonumber \\&\quad \le \Lambda _{k,\delta }\int _\Omega (T_k(n_k)+T_k(p_k) + 2\delta )\textrm{d}x + \frac{7\delta }{2}\int _\Omega |\nabla V_k|^2 \textrm{d}x + \frac{\delta }{2}|\Omega |\Lambda _{k,\delta }. \end{aligned}$$

In view of Poisson’s equation (18), the last term in the first line of (33) can be written as:

$$\begin{aligned} -\big \langle \partial _t(n_k-p_k-D_k),V_k-\bar{V}\big \rangle = -\lambda ^2\langle \partial _t\Delta V_k,V_k-\bar{V}\rangle = \frac{\lambda ^2}{2}\frac{\textrm{d}}{\textrm{d}t}\int _\Omega |\nabla (V_k-\bar{V})|^2 \textrm{d}x. \end{aligned}$$

Integrating (33) over (0, *t*) and taking into account

$$\begin{aligned} \int _\Omega |\nabla V_k|^2 \textrm{d}x \le 2\int _\Omega |\nabla (V_k-\bar{V})|^2 \textrm{d}x + 2\int _\Omega |\nabla \bar{V}|^2 \textrm{d}x \end{aligned}$$

as well as definitions (27) for $E_{k,\delta }$ and (28) for $E_{k,\delta }^I$, we arrive at

$$\begin{aligned}&E_{k,\delta }(n_k,p_k,D_k,V_k)(t) + \frac{1}{2}\int _0^t\int _\Omega \big |\nabla \widetilde{g}_{k,\delta }(n_k) - \sqrt{T_k(n_k)+\delta }\nabla V_k\big |^2 \textrm{d}x\textrm{d}s \\&\qquad + \frac{1}{2}\int _0^t\int _\Omega \big |\nabla \widetilde{g}_{k,\delta }(p_k) + \sqrt{T_k(p_k)+\delta }\nabla V_k\big |^2 \textrm{d}x\textrm{d}s \\&\qquad + \frac{1}{2}\int _0^t\int _\Omega \big |\nabla \widetilde{h}_{k,\delta }(D_k) + (T_{k/(k+1)}(D_k)+\delta )^{1/2}\nabla V_k\big |^2 \textrm{d}x\textrm{d}s \\&\quad \le E_{k,\delta }^I + \Lambda _{k,\delta }\int _0^t \int _\Omega (T_k(n_k)+T_k(p_k) + 2\delta )\textrm{d}x\textrm{d}s\\&\qquad + 7\delta \int _0^t \int _\Omega |\nabla (V_k-\bar{V})|^2 \textrm{d}x\textrm{d}s + 7\delta \int _0^t \int _\Omega |\nabla \bar{V}|^2 \textrm{d}x\textrm{d}s + \frac{\delta }{2}|\Omega |\Lambda _{k,\delta }t. \end{aligned}$$

We conclude from Young’s inequality and Lemma 6 that

$$\begin{aligned} T_k(n_k) \le C + T_k(n_k)^{5/3} \le C + CG_{k,\delta }(n_k), \end{aligned}$$

and consequently, the second term on the right-hand side can be replaced by:

$$\begin{aligned} C_1\Lambda _{k,\delta }\int _0^t E_{k,\delta }(n_k,p_k,D_k,V_k)\textrm{d}s + C(\Omega )(\delta +1)\Lambda _{k,\delta }t. \end{aligned}$$

Thus, applying Gronwall’s lemma,

$$\begin{aligned}&E_{k,\delta }(n_k,p_k,D_k,V_k)(t) + \frac{1}{2}\int _0^t\int _\Omega \big |\nabla \widetilde{g}_{k,\delta }(n_k) - \sqrt{T_k(n_k)+\delta }\nabla V_k\big |^2 \textrm{d}x\textrm{d}s \\&\qquad + \frac{1}{2}\int _0^t\int _\Omega \big |\nabla \widetilde{g}_{k,\delta }(p_k) + \sqrt{T_k(p_k)+\delta }\nabla V_k\big |^2 \textrm{d}x\textrm{d}s \\&\qquad + \frac{1}{2}\int _0^t\int _\Omega \big |\nabla \widetilde{h}_{k,\delta }(D_k) + (T_{k/(k+1)}(D_k)+\delta )^{1/2}\nabla V_k\big |^2 \textrm{d}x\textrm{d}s \\&\quad \le \big (E_{k,\delta }^I + C(\Omega )(\delta +1)\Lambda _{k,\delta }t\big ) \exp (C_1\Lambda _{k,\delta }t). \end{aligned}$$

This proves the lemma. $\square$

### Limit $\delta \rightarrow 0$

We set

$$\begin{aligned} \widetilde{g}_k(s)&= \widetilde{g}_{k,0}(s), \quad G_k(s) = G_{k,0}(s), \quad \widetilde{h}_k(s) = \widetilde{h}_{k,0}(s), \quad H_k(s) = H_{k,0}(s), \\ \mathcal {G}_k(s|\bar{s})&= \mathcal {G}_{k,0}(s|\bar{s}), \quad \mathcal {H}_k(s|\bar{s}) = \mathcal {H}_{k,0}(s|\bar{s}) \end{aligned}$$

and introduce

$$\begin{aligned} E_k(n_k,p_k,D_k,V_k)&= \int _\Omega \bigg (\mathcal {G}_k(n_k|\bar{n}) + \mathcal {G}_k(p_k|\bar{p}) + \mathcal {H}_k(V_k) + \frac{\lambda ^2}{2}|\nabla (V_k-\bar{V})|^2\bigg )\textrm{d}x, \\ E_k^I&= E_k(n^I,p^I,D^I,V^I), \quad \Lambda _k = \Lambda _{k,0}. \end{aligned}$$

#### Lemma 8

(Approximate free energy inequality for $E_k$) Under the assumptions of Lemma 7, it holds for all $0<t<T$ that

34 $$\begin{aligned}&E_{k}(n_k,p_k,D_k,V_k)(t) + \frac{1}{2}\int _0^t\int _\Omega \big |\nabla \widetilde{g}_{k}(n_k) - \sqrt{T_k(n_k)}\nabla V_k\big |^2 \textrm{d}x\textrm{d}s \nonumber \\&\quad + \frac{1}{2}\int _0^t\int _\Omega \big |\nabla \widetilde{g}_{k}(p_k) + \sqrt{T_k(p_k)}\nabla V_k\big |^2 \textrm{d}x\textrm{d}s \nonumber \\&\quad + \frac{1}{2}\int _0^t\int _\Omega \big |\nabla \widetilde{h}_{k}(D_k) + T_{k/(k+1)}(D_k)^{1/2}\nabla V_k\big |^2 \textrm{d}x\textrm{d}s \le C(E_k^I,\Lambda _k,T), \end{aligned}$$

and the constant $C(E_k^I,\Lambda _k,T)\ge 0$ vanishes if $\Lambda _k=0$.

#### Proof

The proof essentially follows from the monotone and dominated convergence theorems as in the proof of Lemma 2.3 of Jourdana et al. (2023). The only difference is the treatment of the functions $\widetilde{g}_{k,\delta }$, $G_{k,\delta }$, $\widetilde{h}_{k,\delta }$, and $H_{k,\delta }$. In fact, by the monotone convergence theorem, $\widetilde{g}_{k,\delta }(n_k)\rightarrow \widetilde{g}_k(n_k)$, $G_{k,\delta }(n_k)\rightarrow G_k(n_k)$, $\widetilde{h}_{k,\delta }(D_k)\rightarrow \widetilde{h}_k(D_k)$, and $H_{k,\delta }(D_k)\rightarrow H_k(D_k)$ a.e. in $\Omega _T$ as $\delta \rightarrow 0$. We derive upper bounds for the limit functions. Let $s>k$. Then, using Lemma 20,

$$\begin{aligned} \widetilde{g}_k(s)&= \int _0^{s}\widetilde{g}'_k(z)\textrm{d}z = \int _0^k\sqrt{z}g'(z)dz + \int _k^{s}k^{1/6}z^{1/3}g'(z)\textrm{d}z \\&\le C\int _0^k(z^{1/6}+z^{-1/2})\textrm{d}z + Ck^{1/6}\int _k^s(1+z^{-2/3})\textrm{d}z \le C(k)(s+1), \end{aligned}$$

and this inequality also holds for any $s\ge 0$. We obtain for $s>k/(k+1)$:

$$\begin{aligned} \widetilde{h}_k(s) = \int _0^{k/(k+1)}\frac{\textrm{d}z}{\sqrt{z}(1-z)} + \int _{k/(k+1)}^s(k+1)\sqrt{\frac{k+1}{k}}\textrm{d}z \le C(k)(s+1), \end{aligned}$$

and this bound holds in fact for all $s\ge 0$. Similar arguments lead to

$$\begin{aligned} G_{k,\delta }(s)\le C(k)(s^2+1), \quad H_{k,\delta }(s)\le C(k)(s^2+1)\quad \text{ for } s\ge 0. \end{aligned}$$

The approximate free energy inequality (30) implies that $n_k,p_k,D_k$ are bounded in $L^2(\Omega _T)$ uniformly in $\delta$. Hence, we can apply the dominated convergence theorem to find that

$$\begin{aligned}&\widetilde{g}_{k,\delta }(n_k)\rightarrow \widetilde{g}_k(n_k), \quad \widetilde{h}_{k,\delta }(D_k)\rightarrow \widetilde{h}_k(D_k) \quad \text{ strongly } \text{ in } L^2(\Omega _T), \\&G_{k,\delta }(n_k)\rightarrow G_k(n_k), \quad H_{k,\delta }(D_k)\rightarrow H_k(D_k) \quad \text{ strongly } \text{ in } L^1(\Omega _T). \end{aligned}$$

The sequence $(\nabla \widetilde{g}_{k,\delta }(n_k)-\sqrt{T_k(n_k)+\delta }\nabla V_k)_\delta$ is uniformly bounded in $L^2(\Omega _T)$. Therefore, there exists a subsequence that converges weakly in $L^2(\Omega _T)$ as $\delta \rightarrow 0$. The previous arguments allow us to identify the weak limit, showing the claim. The other terms in (30) can be treated in a similar way. The limit $\delta \rightarrow 0$ in (30) then proves (34). $\square$

### Uniform Estimates

We first show some inequalities relating *g*, $G_k$, $T_k$, and $\widetilde{g}$.

#### Lemma 9

There exists $C>0$ such that for all $k>1$ and $s>0$,

$$\begin{aligned}&g'(s)\le G_k''(s), \quad s^{5/3}\le C(G_k(s)+1), \quad T_k(s)^{7/6}\le C\widetilde{g}_k(s), \\&\widetilde{g}_k(s)^{10/7}\le C(G_k(s)+1), \quad T_k(s)^{5/3}\le C(G_k(s)+1), \\&\widetilde{h}_k(s)\ge s^{-1/2}, \quad \widetilde{h}'_k(s)\ge 1. \end{aligned}$$

#### Proof

The first inequality follows from $G_k''(s)=g'(s)$ for $0<s\le k$ and $G_k''(s)=(s/k)^{1/3}g'(s)\ge g'(s)$ for $s>k$. Since $g'(s)\sim s^{-1}+s^{-1/3}$ by Lemma 20,

$$\begin{aligned} G_k(s) \ge C\int _{\mathcal {F}_{1/2}(0)}^s\int _{\mathcal {F}_{1/2}(0)}^y(z^{-1}+z^{-1/3}) \textrm{d}z\textrm{d}y \ge C(s^{5/3}-1), \end{aligned}$$

which proves the second inequality. For the third one, let $0<s\le k$. Then, again by Lemma 20,

$$\begin{aligned} \widetilde{g}'_k(z) = \sqrt{z}g'(z) \ge C(z^{-1/2}+z^{1/6}) \ge Cz^{1/6}. \end{aligned}$$

We integrate this inequality over $z\in (0,s)$ to find that $\widetilde{g}_k(s)=\widetilde{g}_k(s)-\widetilde{g}_k(0)\ge Cs^{7/6} = CT_k(s)^{7/6}$. If $s>k$, we compute

$$\begin{aligned} \widetilde{g}_k(s)\ge \int _0^k\frac{yg'(y)}{\sqrt{T_k(y)}}\textrm{d}y = \int _0^k\sqrt{y}g'(y)\textrm{d}y \ge C\int _0^k y^{1/6}\textrm{d}y = Ck^{7/6} = CT_k(s)^{7/6}. \end{aligned}$$

We turn to the fourth inequality. By Lemma 20, we have for $0<s\le k$,

$$\begin{aligned} \widetilde{g}_k(s) = \int _0^s \sqrt{y}g'(y)\textrm{d}y \le C\int _0^s(y^{-1/2}+y^{1/6})\textrm{d}y \le C(s^{7/6}+1). \end{aligned}$$

Furthermore, if $$s>k$$,

$$\begin{aligned} \widetilde{g}_k(s)&= \int _0^k\sqrt{y}g'(y)\textrm{d}y + \int _k^s k^{1/6}y^{1/3}g'(y)\textrm{d}y \\&\le C(k^{1/2}+k^{7/6}) + Ck^{1/6}(s^{1/3}+s) \le C(s^{7/6}+1), \end{aligned}$$

and the conclusion follows after raising the inequality to the power 10/7 and using the second inequality. The fifth inequality follows from the third and fourth ones since $T_k(s)^{5/3}\le C\widetilde{g}_k(s)^{10/7}\le C(G_k(s)+1)$.

To estimate $\widetilde{h}'$, we observe that $h'(s)=1/(s(1-s))$ and hence $\widetilde{h}'_k(s)=1/(\sqrt{s}(1-s))\ge s^{-1/2}$ for $s<k/(k+1)$ and $\widetilde{h}'_k(s)=(1+k)/k\ge s^{-1}\ge s^{-1/2}$ for $k/(k+1)\le s<1$. Moreover, in both cases, $\widetilde{h}'(s)\ge 1$. This proves the inequalities for $\widetilde{h}'_k$. $\square$

The previous lemma and the approximate energy inequality (34) lead to the following a priori estimates.

#### Lemma 10

(Uniform estimates I) There exists a constant $C>0$ such that for all $k\in {{\mathbb {N}}}$,

$$\begin{aligned}&\Vert T_k(n_k)\Vert _{L^\infty (0,T;L^{5/3}(\Omega ))} + \Vert T_k(p_k)\Vert _{L^\infty (0,T;L^{5/3}(\Omega ))} \le C, \\&\Vert n_k\Vert _{L^\infty (0,T;L^{5/3}(\Omega ))} + \Vert p_k\Vert _{L^\infty (0,T;L^{5/3}(\Omega ))} \le C, \\&\Vert \widetilde{g}_k(n_k)\Vert _{L^\infty (0,T;L^{10/7}(\Omega ))} + \Vert \widetilde{g}_k(p_k)\Vert _{L^\infty (0,T;L^{10/7}(\Omega ))} \le C, \\&\Vert \sqrt{T_k(n_k)}\nabla V_k\Vert _{L^\infty (0,T;L^{5/4}(\Omega ))} + \Vert \sqrt{T_k(p_k)}\nabla V_k\Vert _{L^\infty (0,T;L^{5/4}(\Omega ))} \le C, \\&\Vert \nabla \widetilde{g}_k(n_k)\Vert _{L^2(0,T;L^{5/4}(\Omega ))} + \Vert \nabla \widetilde{g}_k(p_k)\Vert _{L^2(0,T;L^{5/4}(\Omega ))} \le C, \\&\Vert \nabla n_k\Vert _{L^2(0,T;L^{5/4}(\Omega ))} + \Vert \nabla p_k\Vert _{L^2(0,T;L^{5/4}(\Omega ))} \le C, \\&\Vert \nabla \widetilde{h}_k(D_k)\Vert _{L^2(\Omega _T)} + \Vert (T_{k/(k+1)}(D_k))^{1/2}\nabla V_k\Vert _{L^\infty (0,T;L^2(\Omega ))}\le C, \\&\Vert \nabla D_k\Vert _{L^2(\Omega _T)} + \Vert \nabla \sqrt{D_k}\Vert _{L^2(\Omega _T)} \le C. \end{aligned}$$

#### Proof

The approximate energy inequality (34) shows that $(G_k(n_k))$ and $(G_k(p_k))$ are bounded in $L^\infty (0,T;L^1(\Omega ))$. By Lemma 9, this yields a uniform bound for $T_k(n_k)$, $T_k(p_k)$ and $n_k$, $p_k$ in $L^\infty (0,T;L^{5/3}(\Omega ))$ and for $\widetilde{g}_k(n_k)$, $\widetilde{g}_k(p_k)$ in $L^\infty (0,T;L^{10/7}(\Omega ))$. The energy estimate (34) implies a uniform bound for $\nabla V_k$ in $L^\infty (0,T;L^2(\Omega ))$. Consequently, using Hölder’s inequality,

$$\begin{aligned} \Vert \sqrt{T_k(n_k)}\nabla V_k\Vert _{L^\infty (0,T;L^{5/4}(\Omega ))} \!\le \! \Vert \sqrt{T_k(n_k)}\Vert _{L^\infty (0,T;L^{10/3}(\Omega ))} \Vert \nabla V_k\Vert _{L^\infty (0,T;L^2(\Omega ))} \!\le \! C, \end{aligned}$$

and similarly for $\sqrt{T_k(p_k)}\nabla V_k$. Then we deduce from the $L^2(\Omega _T)$ bound for $\nabla \widetilde{g}_k(n_k)-\sqrt{T_k(n_k)}\nabla V_k$ that $\nabla \widetilde{g}_k(n_k)$ is uniformly bounded in $L^2(0,T;L^{5/4}(\Omega ))$. It follows from Lemma 20 that $\widetilde{g}_k'(s)=\sqrt{s}g'(s)\ge C(s^{1/6}+s^{-1/2})\ge C$ for $0<s\le k$ and $\widetilde{g}_k'(s) = k^{1/6}s^{1/3}g'(s)\ge k^{1/6}(1+s^{-2/3})\ge 1$ for $s>k$. Thus, $\widetilde{g}_k'$ is bounded from below by a positive constant. Then, the bounds for $\nabla \widetilde{g}_k(n_k)$ and $\nabla \widetilde{g}_k(p_k)$ imply the same bounds for $\nabla n_k$ and $\nabla p_k$.

We turn to the estimates for $D_k$. Since $T_{k/(k+1)}(D_k)<1$, we infer from the energy inequality (34) that $(T_{k/(k+1)}(D_k)^{1/2}\nabla V_k)$ is bounded in $L^\infty (0,T;L^2(\Omega ))$ and consequently, $\nabla \widetilde{h}_k(D_k)$ is uniformly bounded in $L^2(\Omega _T)$. We deduce from $\widetilde{h}_k'(s)\ge s^{-1/2}$ and $\widetilde{h}_k'(s)\ge 1$ for $s>0$ (see Lemma 9) that $\nabla \sqrt{D_k}$ and $\nabla D_k$ are uniformly bounded in $L^2(\Omega _T)$. $\square$

We can improve the regularity stated in Lemma 10 by using the inequality $T_k(s)^{7/6}\le C\widetilde{g}_k(s)$ and an iteration argument.

#### Lemma 11

(Uniform estimates II) Let $q,r\le \infty$ if $d=1$, $q,r<\infty$ if $d=2$, and $q<8$, $r<24/7$ if $d=3$. There exists a constant $C>0$ such that for all $k\in {{\mathbb {N}}}$,

$$\begin{aligned}&\Vert \sqrt{T_k(n_k)}\Vert _{L^{14/3}(0,T;L^q(\Omega ))} + \Vert \sqrt{T_k(n_k)}\Vert _{L^{14/3}(0,T;L^q(\Omega ))} \le C, \\&\Vert \sqrt{T_k(n_k)}\nabla V_k\Vert _{L^{14/3}(0,T;L^{r}(\Omega ))} + \Vert \sqrt{T_k(n_k)}\nabla V_k\Vert _{L^{14/3}(0,T;L^{r}(\Omega ))} \le C,\\&\Vert \widetilde{g}_k(n_k)\Vert _{L^2(0,T;L^{r}(\Omega ))} + \Vert \widetilde{g}_k(p_k)\Vert _{L^2(0,T;L^{r}(\Omega ))} \le C,\\&\Vert \nabla \widetilde{g}_k(n_k)\Vert _{L^2(0,T;L^{2q/(2+q)}(\Omega ))} + \Vert \nabla \widetilde{g}_k(p_k)\Vert _{L^2(0,T;L^{2q/(2+q)}(\Omega ))} \le C,\\&\Vert \nabla n_k\Vert _{L^2(0,T;L^{2q/(2+q)}(\Omega ))} + \Vert \nabla p_k\Vert _{L^2(0,T;L^{2q/(2+q)}(\Omega ))} \le C \end{aligned}$$

Observe that $2q/(2+q)<8/5$ if $d=3$.

#### Proof

We assume that $(\sqrt{T_k(n_k)})$ is bounded in $L^{14/3}(0,T;L^{q_{m}}(\Omega ))$ and $(\widetilde{g}_k(n_k))$ is bounded in $L^2(0,T;L^{r_{m}}(\Omega ))$ for some numbers $q_m$, $r_m\ge 1$ with $m\in {{\mathbb {N}}}$. By Lemma 10, we have $q_1=10/3$ and $r_1=10/7$. We estimate

35 $$\begin{aligned}&\Vert \sqrt{T_k(n_k)}\nabla V_k\Vert _{L^{14/3}(0,T;L^a(\Omega ))} \le \Vert \sqrt{T_k(n_k)}\Vert _{L^{14/3}(0,T;L^{q_m}(\Omega ))} \Vert \nabla V_k\Vert _{L^\infty (0,T;L^2(\Omega ))} \le C, \end{aligned}$$

where $1/a = 1/q_m+1/2$. This shows that

36 $$\begin{aligned} \Vert \nabla \widetilde{g}_k(n_k)\Vert _{L^2(0,T;L^a(\Omega ))}&\le \Vert \nabla \widetilde{g}_k(n_k) -\sqrt{T_k(n_k)}\nabla V_k\Vert _{L^2(0,T;L^a(\Omega ))} \nonumber \\&\quad + \Vert \sqrt{T_k(n_k)}\nabla V_k\Vert _{L^{2}(0,T;L^a(\Omega ))} \le C. \end{aligned}$$

Then, the continuous embedding $W^{1,a}(\Omega )\hookrightarrow L^{r_{m+1}}(\Omega )$ with $1/r_{m+1}=1/a-1/d=1/q_m+1/2-1/d$ implies that $\widetilde{g}_k(n_k)$ is uniformly bounded in $L^2(0,T;L^{r_{m+1}}(\Omega ))$. We deduce from $\sqrt{T_k(s)}\le C\widetilde{g}_k(s)^{3/7}$ (see Lemma 9) that $\sqrt{T_k(n_k)}$ is uniformly bounded in $L^{14/3}(0,T;L^{q_{m+1}}(\Omega ))$, where $q_{m+1}=7r_{m+1}/3$. This leads to the recursion

$$\begin{aligned} \frac{1}{q_{m+1}} = \frac{3}{7}\frac{1}{r_{m+1}} = \frac{3}{7}\bigg (\frac{1}{q_m}+\frac{1}{2}-\frac{1}{d}\bigg ). \end{aligned}$$

The sequence $(1/q_m)$ is nonincreasing (if $d\le 4$) and bounded from below. Thus, it possesses the limit $q^*$ that satisfies

$$\begin{aligned} \frac{1}{q^*} = \frac{3}{7}\bigg (\frac{1}{q^*}+\frac{1}{2}-\frac{1}{d}\bigg ) \quad \text{ and } \text{ hence }\quad q^* = \frac{8d}{3(d-2)}. \end{aligned}$$

We can perform this recursion only a finite number of times as otherwise the powers of the embedding constant may diverge. Thus, $q<q^*=8$ if $d=3$ and, since $q_{m+1}=7q_m/3\rightarrow \infty$ as $m\rightarrow \infty$, $q<\infty$ if $d=2$. Furthermore, $1/r=1/q+1/2-1/d$, which gives $r<24/7$ if $d=3$ and $r<\infty$ if $d=2$.

The uniform bound for $\sqrt{T_k(n_k)}\nabla V_k$ follows from (35) because of $1/a=1/q+1/2=(2+q)/(2q)$. Estimate (36) then implies the bound for $\nabla \widetilde{g}_k(n_k)$. We have shown in the proof of Lemma 10 that $\widetilde{g}_k'$ is bounded from below by a positive constant such that $C|\nabla n_k|\le |\widetilde{g}'_k(n_k)\nabla n_k|=|\nabla \widetilde{g}_k(n_k)|$, proving the last uniform bound. Finally, the estimates for $p_k$ are proved analogously. $\square$

The following bounds are needed for the Aubin–Lions lemma.

#### Lemma 12

(Uniform estimates III) Under the assumptions of Lemma 11, there exists a constant $C>0$ such that for all $k\in {{\mathbb {N}}}$,

$$\begin{aligned}&\Vert n_k\Vert _{L^{2}(0,T;W^{1,2q/(2+q)}(\Omega ))} + \Vert p_k\Vert _{L^{2}(0,T;W^{1,2q/(2+q)}(\Omega ))} \le C, \\&\Vert \partial _t n_k\Vert _{L^{7/5}(0,T;W_D^{1,2q/(q+4)}(\Omega )')} + \Vert \partial _t p_k\Vert _{L^{7/5}(0,T;W_D^{1,2q/(q+4)}(\Omega )')} \le C, \\&\Vert D_k\Vert _{L^2(0,T;H^1(\Omega ))} + \Vert \partial _t D_k\Vert _{L^2(0,T;H^1(\Omega )')} \le C. \end{aligned}$$

#### Proof

The bound for $n_k$ follows from the gradient bounds in Lemma 11 and the bound for $n_k$ in $L^\infty (0,T;L^{5/3}(\Omega ))$, which is a consequence of the energy inequality (34) and the inequality $s^{5/3}\le C(G_k(s)+1)$ from Lemma 9. By the chain rule (24) (for $\delta =0$), the evolution equation for $n_k$ reads as:

$$\begin{aligned} \partial _t n_k = \operatorname {div}\big [\sqrt{T_k(n_k)}\big (\nabla \widetilde{g}_k(n_k) - \sqrt{T_k(n_k)}\nabla V_k\big )\big ]. \end{aligned}$$

The term $\sqrt{T_k(n_k)}$ is uniformly bounded in $L^{14/3}(0,T;L^q(\Omega ))$, while $\nabla \widetilde{g}_k(n_k) - \sqrt{T_k(n_k)}\nabla V_k$ is uniformly bounded in $L^2(0,T;L^{2q/(2+q)}(\Omega ))$. Hence, $(\partial _t n_k)$ is bounded in $L^{7/5}(0,T;$ $L^{2q/(4+q)}(\Omega ))$ (we choose $q\ge 4$ to guarantee that $2q/(4+q)\ge 1$). The estimates for $p_k$ are shown in a similar way.

We turn to the bounds for $D_k$. We know from the energy inequality (34) that $(H_k(D_k))$ is bounded in $L^\infty (0,T;L^1(\Omega ))$ and from Lemma 10 that $(\nabla D_k)$ is bounded in $L^2(\Omega _T)$. Proceeding as in the proof of Lemma 9, we infer that $S_k^2/T_{k/(k+1)}$ is bounded from below by a positive constant. This implies that $H_k(D_k)\ge CD_k^2$, which yields a bound for $(D_k)$ in $L^\infty (0,T;L^2(\Omega ))$. This shows that $(D_k)$ is bounded in $L^2(0,T;H^1(\Omega ))$. Finally, since $(T_{k/(k+1)}(D_k))$ is bounded in $L^\infty (\Omega _T)$, the sequence

$$\begin{aligned} (\nabla \widetilde{h}_k(D_k)+T_{k/(k+1)}(D_k)^{1/2}\nabla V_k) \end{aligned}$$

is bounded in $L^2(\Omega _T)$. Therefore,

$$\begin{aligned} \partial _t D_k = \operatorname {div}\big [T_{k/(k+1)}(D_k)^{1/2}\big (\nabla \widetilde{h}_k(D_k) + T_{k/(k+1)}(D_k)^{1/2}\nabla V_k\big )\big ] \end{aligned}$$

is uniformly bounded in $L^2(0,T;H^1(\Omega )')$, finishing the proof. $\square$

### Limit $k\rightarrow \infty$ in the Equations for $n_k$ and $p_k$

Lemma 12 and the compact embedding $W^{1,2q/(2+q)}(\Omega )\hookrightarrow L^r(\Omega )$ for $r<24/7$ (if $d\le 3$) allow us to apply the Aubin–Lions lemma to infer the existence of a subsequence that is not relabeled such that, as $k\rightarrow \infty$,

$$\begin{aligned} (n_k,p_k)\rightarrow (n,p)&\quad \text{ strongly } \text{ in } L^2(0,T;L^{r}(\Omega ))^2, \\ D_k\rightarrow D&\quad \text{ strongly } \text{ in } L^2(\Omega _T), \\ (\nabla n_k,\nabla p_k)\rightharpoonup (\nabla n,\nabla p)&\quad \text{ weakly } \text{ in } L^2(0,T;L^{2q/(2+q)}(\Omega )), \\ (\partial _t n_k,\partial _t p_k)\rightharpoonup (\partial _t n,\partial _t p)&\quad \text{ weakly } \text{ in } L^{7/5}(0,T;W_D^{1,2q/(q+4)}(\Omega )')^2, \\ \partial _t D_k \rightharpoonup \partial _t D&\quad \text{ weakly } \text{ in } L^2(0,T;H^1(\Omega )'). \end{aligned}$$

The $L^\infty (0,T;L^{5/3}(\Omega ))$ bound for $n_k$ from Lemma 10 implies that

$$\begin{aligned} \Vert T_k(n_k)-n_k\Vert _{L^1(\Omega _T)} = \int _0^T\int _{\{n_k>k\}}|k-n_k|\textrm{d}x\textrm{d}t \le \int _0^T\int _\Omega \frac{n_k^{5/3}}{k^{2/3}}\textrm{d}x\textrm{d}t \le \frac{C}{k^{2/3}}\rightarrow 0. \end{aligned}$$

This shows that $\sqrt{T_k(n_k)}-\sqrt{n_k}\rightarrow 0$ strongly in $L^2(\Omega _T)$ and in particular $\sqrt{T_k(n_k)}\rightarrow \sqrt{n}$ strongly in $L^2(\Omega _T)$. In fact, in view of the $L^\infty (0,T;L^{5/3}(\Omega ))$ bound for $n_k$, we even have strong convergence for $\sqrt{T_k(n_k)}$ in $L^s(\Omega _T)$ for any $s<10/3$. The $L^2(\Omega _T)$ bound for $\nabla V_k$ shows that $\nabla V_k\rightharpoonup \nabla V$ weakly in $L^2(\Omega _T)$. Hence,

$$\begin{aligned} \sqrt{T_k(n_k)}\nabla V_k\rightharpoonup \sqrt{n}\nabla V \quad \text{ weakly } \text{ in } L^1(\Omega _T). \end{aligned}$$

Thanks to Lemma 11, this convergence also holds in $L^{14/3}(0,T;L^{r}(\Omega ))$ with $r<8/5$. Then, since $s<10/3$ and $q<8$ can be chosen in such a way that $1/s+(2+q)/(2q)<1$,

37 $$\begin{aligned} T_k(n_k)\nabla V_k = \sqrt{T_k(n_k)}\cdot \sqrt{T_k(n_k)}\nabla V_k \rightharpoonup n\nabla V\quad \text{ weakly } \text{ in } L^1(\Omega _T). \end{aligned}$$

Similarly, we have $T_k(p_k)\nabla V_k\rightharpoonup T_k(p_k)\nabla V_k$ weakly in $L^1(\Omega _T)$.

Next, we prove the weak convergence of $(\sqrt{T_k(n_k)}\nabla \widetilde{g}_k(n_k))$.

#### Lemma 13

It holds that, up to a subsequence,

$$\begin{aligned} \sqrt{T_k(n_k)}\big (\nabla \widetilde{g}_k(n_k)-\sqrt{T_k(n_k)}\nabla V_k\big ) \rightharpoonup n\nabla g(n)-n\nabla V&\quad \text{ weakly } \text{ in } L^1(\Omega _T), \\ \sqrt{T_k(p_k)}\big (\nabla \widetilde{g}_k(p_k)+\sqrt{T_k(p_k)}\nabla V_k\big ) \rightharpoonup p\nabla g(p) + p\nabla V&\quad \text{ weakly } \text{ in } L^1(\Omega _T). \end{aligned}$$

#### Proof

First, we show that $\sqrt{T_k(n_k)}\widetilde{g}_k'(n_k)$ converges strongly. To this end, we estimate

38 $$\begin{aligned}&\big \Vert \sqrt{T_k(n_k)}\widetilde{g}_k'(n_k) - ng'(n) \big \Vert _{L^2(0,T;L^{5}(\Omega ))} \nonumber \\&\quad \le \big \Vert \sqrt{T_k(n_k)}\widetilde{g}_k'(n_k) - n_kg'(n_k) \big \Vert _{L^2(0,T;L^{5}(\Omega ))} + \Vert n_kg'(n_k)-ng'(n)\Vert _{L^2(0,T;L^{5}(\Omega ))}. \end{aligned}$$

The first integrand vanishes on $\{n_k\le k\}$. Therefore, on the set $\{n_k>k\}$, using Lemma 20,

$$\begin{aligned}&\big |\sqrt{T_k(n_k)}\widetilde{g}_k'(n_k) - n_kg'(n_k)\big |^5 = |(n_k^{2/3}-k^{2/3})n_k^{1/3}g'(n_k)|^{5} \\&\quad \le |n_kg'(n_k)|^{5} \le C|n_k(n_k^{-1}+n_k^{-1/3})|^{5} \le C(1+n_k^{10/3}) \le C\bigg (\frac{n_k}{k}+\frac{n_k^{10/3+\varepsilon }}{k^{\varepsilon }}\bigg ) \end{aligned}$$

for $\varepsilon >0$ such that $10/3+\varepsilon \le r<24/7$, which shows that

$$\begin{aligned} \big \Vert \sqrt{T_k(n_k)}\widetilde{g}_k'(n_k) - n_kg'(n_k) \big \Vert _{L^2(0,T;L^{5}(\Omega ))}^2 \le \frac{C}{k^{2\varepsilon }}\int _0^T\big (\Vert n_k\Vert _{L^1(\Omega )} + \Vert n_k\Vert _{L^r(\Omega )}^{r}\big )^{2/5}dt\rightarrow 0, \end{aligned}$$

since $(n_k)$ is bounded in $L^2(0,T;L^r(\Omega ))$. We show in Lemma 21 that $|(zg'(z))'|=|g'(z)+zg''(z)|$ is bounded for $z>0$. Hence,

$$\begin{aligned} |n_kg'(n_k)-ng'(n)|^r = \bigg |\int _{n_k}^n\frac{\textrm{d}}{\textrm{d}z}(zg'(z))\textrm{d}z\bigg |^r \le C|n_k-n|^r. \end{aligned}$$

Then, the strong convergence $n_k\rightarrow n$ in $L^2(0,T;L^r(\Omega ))$ implies that

$$\begin{aligned} \Vert n_kg'(n_k)-ng'(n)\Vert _{L^2(0,T;L^r(\Omega ))}\rightarrow 0. \end{aligned}$$

We conclude from (38) that

$$\begin{aligned} \sqrt{T_k(n_k)}\widetilde{g}_k'(n_k) \rightarrow ng'(n) \quad \text{ strongly } \text{ in } L^2(0,T;L^r(\Omega )). \end{aligned}$$

Consequently, the weak convergence $\nabla n_k\rightharpoonup \nabla n$ in $L^2(0,T;L^{2q/(2+q)}(\Omega ))$ gives

$$\begin{aligned} \sqrt{T_k(n_k)}\nabla \widetilde{g}_k(n_k) = \sqrt{T_k(n_k)}\widetilde{g}_k'(n_k)\nabla n_k \rightharpoonup ng'(n)\nabla n = n\nabla g(n) \end{aligned}$$

weakly in $$L^1(\Omega 
_T)$$. Combining this result with (37) shows that

$$\begin{aligned} \sqrt{T_k(n_k)}\big (\nabla \widetilde{g}_k(n_k) - \sqrt{T_k(n_k)} \nabla V_k\big ) \rightharpoonup n\nabla g(n)-n\nabla V \quad \text{ weakly } \text{ in } L^1(\Omega _T). \end{aligned}$$

The limit for the term $(\sqrt{T_k(p_k)}\nabla \widetilde{g}_k(p_k))$ is shown analogously, which ends the proof. $\square$

We need the convergence of $(\nabla \widetilde{g}(n_k))$ and $(\nabla \widetilde{g}(p_k))$ to pass to the limit in the energy inequality.

#### Lemma 14

It holds that

$$\begin{aligned} (\nabla \sqrt{n_k},\nabla \sqrt{p_k})\rightharpoonup (\nabla \sqrt{n},\nabla \sqrt{p})&\quad \text{ weakly } \text{ in } L^2(0,T;L^{2q/(2+q)}(\Omega ))^2, \\ (\nabla \widetilde{g}_k(n_k),\nabla \widetilde{g}_k(p_k)) \rightharpoonup (\sqrt{n}\nabla g(n),\sqrt{p}\nabla g(p))&\quad \text{ weakly } \text{ in } L^1(\Omega _T)^2. \end{aligned}$$

#### Proof

It follows from Lemma 20 that $\widetilde{g}'_k(s)=\sqrt{s}g'(s)\ge C(s^{-1/2}+s^{1/6})\ge Cs^{-1/2}$ for $s<k$ and $\widetilde{g}'_k(s)=k^{-1/6}s^{1/3}g'(s)\ge CC(s/k)^{1/6}(s^{-5/6}+s^{-1/2})\ge Cs^{-1/2}$ for $s>k$. Then, the uniform bound for $\nabla \widetilde{g}_k(n_k)$ in Lemma 11 implies directly the bound for $\nabla \sqrt{n_k}$ in $L^2(0,T;L^{2q/(2+q)}(\Omega ))$, showing the first statement. The limit for $\nabla \sqrt{p_k}$ is shown analogously. Repeating the proof of Lemma 13 with $\sqrt{n_k}$ instead of $\sqrt{T_k(n_k)}$, we obtain the convergence

$$\begin{aligned} \sqrt{n_k}\widetilde{g}'_k(n_k)\rightarrow ng'(n) \quad \text{ strongly } \text{ in } L^2(0,T;L^r(\Omega )). \end{aligned}$$

We combine this result with the weak convergence of $(\nabla \sqrt{n_k})$ to conclude that

$$\begin{aligned} \nabla \widetilde{g}_k(n_k)=2\sqrt{n_k}\widetilde{g}_k'(n_k)\nabla \sqrt{n_k} \rightharpoonup 2ng'(n)\nabla \sqrt{n} = \sqrt{n}\nabla g(n) \quad \text{ weakly } \text{ in } L^1(\Omega _T), \end{aligned}$$

using that $(2+q)/(2q)+1/r<1$ if we choose $q<8$ and $r<24/7$ sufficiently large. $\square$

### Limit $k\rightarrow \infty$ in the Equation for $D_k$

We prove the convergence of the terms in the equation for $D_k$. First, we consider $T_{k/(k+1)}(D_k)^{1/2}\nabla \widetilde{h}_k(D_k)=T_{k/(k+1)}(D_k)^{1/2}\widetilde{h}'_k(D_k)\nabla D_k$. We introduce the functions

$$\begin{aligned} L(s)&= -\log (1-s) \quad \text{ for } 0\le s<1, \\ L_k(s)&= {\left\{ \begin{array}{ll} -\log (1-s) & \text{ for } 0\le s\le k/(k+1), \\ (k+1)s-k+\log (k+1) & \text{ for } s>k/(k+1). \end{array}\right. } \end{aligned}$$

They satisfy the property $L'_k(D_k)=T_{k/(k+1)}(D_k)^{1/2}\widetilde{h}'_k(D_k)$ for all $0\le s<1$. Moreover, $L_k$ is nondecreasing, $L_k\le L_{k+1}$ on [0, 1), and $L_k$ converges to *L* locally uniformly on [0, 1). The aim is to derive uniform bounds for $L_k(D_k)$ and to identify its weak limit.

#### Lemma 15

There exists a constant $C>0$ such that

$$\begin{aligned} \Vert L_k(D_k)\Vert _{L^2(0,T;H^1(\Omega ))}\le C. \end{aligned}$$

#### Proof

The gradient bound follow immediately from $L'_k(s)<\widetilde{h}'_k(s)$ for $0<s<1$ and $|\nabla L_k(D_k)|=L'_k(D_k)|\nabla D_k|\le \widetilde{h}'_k(D_k)|\nabla D_k|=|\nabla \widetilde{h}_k(D_k)|$, showing that $(\nabla L_k(D_k))$ is bounded in $L^2(\Omega _T)$. By mass conservation and Assumption (A4), we have

$$\begin{aligned} \frac{1}{\textrm{m}(\Omega )}\int _\Omega D_k \textrm{d}x = \frac{1}{\textrm{m}(\Omega )}\int _\Omega D^I \textrm{d}x = D_\Omega ^I < 1. \end{aligned}$$

Thus, the conditions of Lemma 22 in Appendix B are satisfied, and we infer from the gradient bound that $L_k(D_k)$ is bounded in $L^2(\Omega _T)$. Lemma 22 now finishes the proof. $\square$

The uniform bound in Lemma 15 implies the existence of a subsequence such that

$$\begin{aligned} L_k(D_k)\rightharpoonup L^* \quad \text{ weakly } \text{ in } L^2(\Omega _T) \text{ as } k\rightarrow \infty . \end{aligned}$$

The next aim is to identify $L^*=L(D)$, where we recall that *D* is the strong $L^2(\Omega _T)$ limit of $(D_k)$.

First, we claim that $L(D)\in L^2(\Omega _T)$ and $D<1$ a.e. in $\Omega _T$. Indeed, we have $D_\ell \rightarrow D$ a.e. in $\Omega _T$ as $\ell \rightarrow \infty$, up to a subsequence. Hence, since $L_k$ is continuous and $L_k\le L_{k+1}$, for any $k\in {{\mathbb {N}}}$,

$$\begin{aligned}&\int _0^T \int _\Omega L_k(D)^2 \textrm{d}x\textrm{d}t = \int _0^T\int _\Omega \lim _{\ell \rightarrow \infty }L_k(D_\ell )^2 \textrm{d}x\textrm{d}t = \int _0^T\int _\Omega \liminf _{\ell \rightarrow \infty }L_k(D_\ell )^2 \textrm{d}x\textrm{d}t \\&\quad \le \int _0^T\int _\Omega \liminf _{\ell \rightarrow \infty }L_\ell (D_\ell )^2 \textrm{d}x\textrm{d}t \le \liminf _{\ell \rightarrow \infty }\int _0^T\int _\Omega L_\ell (D_\ell )^2 \textrm{d}x\textrm{d}t, \end{aligned}$$

where the last step follows from Fatou’s lemma. The last integral is uniformly bounded. Therefore, $(L_k(D))$ is bounded in $L^2(\Omega _T)$, and again by Fatou’s lemma,

$$\begin{aligned} \int _0^T\int _\Omega L(D)^2 \textrm{d}x\textrm{d}t \le \liminf _{k\rightarrow \infty } \int _0^T\int _\Omega L_k(D)^2 \textrm{d}x\textrm{d}t \le C. \end{aligned}$$

The $L^2(\Omega _T)$-bound for $L(D)=-\log (1-D)$ implies that $D<1$ a.e.

To identify $L^*$ with *L*(*D*), we set

$$\begin{aligned} D_\eta := (1-\eta )D+\eta , \quad D_{k,\eta } :=(1-\eta )D_k+\eta \quad \text{ for } 0<\eta <1. \end{aligned}$$

Because of the strong convergence of $D_k$, we clearly have $D_{k,\eta }\rightarrow D_\eta$ strongly in $L^2(\Omega _T)$ for any fixed $0<\eta <1$. The proof of Lemma 15 shows that $(L_k(D_{k,\eta }))$ is bounded in $L^2(0,T;H^1(\Omega ))$, and the previous arguments imply that $L(D_\eta )\in L^2(\Omega _T)$. Furthermore, let $\psi :\Omega _T\rightarrow [0,1]$ be a smooth function and set

$$\begin{aligned} D_{\eta ,\psi } := (1 - \eta \psi )D + \eta \psi = D + (1-D)\eta \psi , \quad \text{ for } 0< \eta < 1. \end{aligned}$$

Since $D_{\eta ,\psi } \le D_\eta$, the monotonicity of *L* implies that $L(D_{\eta ,\psi }) \in L^2(\Omega _T)$. With these preparations, we can prove that $L^*=L(D)$.

#### Lemma 16

It holds that $L^*=L(D)$ and

$$\begin{aligned} L_k(D_k)\rightharpoonup L(D)\quad \text{ weakly } \text{ in } L^2(0,T;H^1(\Omega )). \end{aligned}$$

#### Proof

The proof is based on the monotonicity of $L_k$ (Minty trick). We pass to the limit $k\rightarrow \infty$ in

$$\begin{aligned} 0 \le \int _0^T\int _\Omega (L_k(D_{\eta ,\psi })-L_k(D_k))(D_{\eta ,\psi }-D_k)\textrm{d}x\textrm{d}t, \end{aligned}$$

leading to

$$\begin{aligned} 0 \le \frac{1}{\eta }\int _0^T\int _\Omega (L(D_{\eta ,\psi })-L^*) (D_{\eta ,\psi }-D)\textrm{d}x\textrm{d}t = \int _0^T\int _\Omega (L(D_{\eta ,\psi })-L^*)(1-D)\psi \textrm{d}x\textrm{d}t. \end{aligned}$$

By dominated convergence, the limit $\eta \rightarrow 0$ gives

39 $$\begin{aligned} 0 \le \int _0^T\int _\Omega (L(D)-L^*)(1-D)\psi \textrm{d}x\textrm{d}t. \end{aligned}$$

Next, we show the inverse inequality. The monotonicity $L_k\le L_{k+1}$, the weak convergence of $(L_k(D_k))$, and Fatou’s lemma yield the following inequalities:

$$\begin{aligned}&\int _0^T \int _\Omega (1-D)\psi L_k(D)\textrm{d}x\textrm{d}t = \int _0^T\int _\Omega (1-D)\psi \lim _{\ell \rightarrow \infty }L_k(D_\ell )\textrm{d}x\textrm{d}t \\&\quad \le \int _0^T\int _\Omega (1-D)\psi \liminf _{\ell \rightarrow \infty }L_\ell (D_\ell )\textrm{d}x\textrm{d}t \le \liminf _{\ell \rightarrow \infty }\int _0^T\int _\Omega (1-D)\psi L_\ell (D_\ell )\textrm{d}x\textrm{d}t \\&\quad \le \int _0^T\int _\Omega (1-D)\psi L^*\textrm{d}x\textrm{d}t. \end{aligned}$$

It follows from dominated convergence that

$$\begin{aligned} \int _0^T\int _\Omega (1-D)\psi L(D)\textrm{d}x\textrm{d}t \le \int _0^T\int _\Omega (1-D)\psi L^*\textrm{d}x\textrm{d}t, \end{aligned}$$

or equivalently,

$$\begin{aligned} \int _0^T\int _\Omega (L(D)-L^*)(1-D)\psi \textrm{d}x\textrm{d}t \le 0. \end{aligned}$$

Together with (39), this shows that

$$\begin{aligned} \int _0^T\int _\Omega (L(D)-L^*)(1-D)\psi \textrm{d}x\textrm{d}t = 0. \end{aligned}$$

Since $\psi$ is arbitrary, we find that $(L(D)-L^*)(1-D)=0$ a.e. It follows from $D<1$ a.e. that $L(D)=L^*$ a.e. Hence, by Lemma 15, $L_k(D_k) \rightharpoonup L(D)$ weakly in $L^2(0,T;H^1(\Omega ))$. $\square$

The previous lemma implies that

40 $$\begin{aligned}&T_{k/(k+1)}(D_k)^{1/2}\nabla \widetilde{h}_k(D_k) = T_{k/(k+1)}(D_k)^{1/2}\widetilde{h}'_k(D_k)\nabla D_k = L_k'(D_k)\nabla D_k \nonumber \\&\quad = \nabla L_k(D_k)\rightharpoonup \nabla L(D) = -\nabla \log (1-D) = D\nabla h(D) \quad \text{ weakly } \text{ in } L^2(\Omega _T). \end{aligned}$$

The next step is the convergence of $(T_{k/(k+1)}(D_k)\nabla V_k)$ and the identification of the limit.

Set $T_1(s)=\max \{0,\min \{1,s\}\}$. We deduce from

$$\begin{aligned} |T_{k/(k+1)}(D_k)-T_1(D)|&= |T_{k/(k+1)}(D_k)-T_{k/(k+1)}(D)| + |T_{k/(k+1)}(D)-T_1(D)| \\&\le |D_k-D| + \frac{1}{k+1} \end{aligned}$$

and the strong convergence of $(D_k)$ that

$$\begin{aligned} \int _0^T\int _\Omega |T_{k/(k+1)}(D_k)-T_1(D)|^2 \textrm{d}x\textrm{d}t \le 2\int _0^T\int _\Omega |D_k-D|^2 \textrm{d}x\textrm{d}t + \frac{C}{(k+1)^2}\rightarrow 0. \end{aligned}$$

The property $D<1$ a.e. implies that $T_1(D)=D$ and thus $T_{k/(k+1)}(D_k)\rightarrow D$ strongly in $L^2(\Omega _T)$. We deduce from the convergence of $\nabla V_k\rightharpoonup \nabla V$ weakly in $L^2(\Omega _T)$ that $T_{k/(k+1)}(D_k)\nabla V_k\rightharpoonup D\nabla V$ weakly in $L^1(\Omega _T)$. We know from Lemma 10 that $T_{k/(k+1)}(D_k)\nabla V_k$ $=T_{k/(k+1)}(D_k)^{1/2}\cdot T_{k/(k+1)}(D_k)^{1/2}\nabla V_K)$ is uniformly bounded in $L^\infty (0,T;L^2(\Omega ))$. This yields the convergence

$$\begin{aligned} T_{k/(k+1)}(D_k)\nabla V_k \rightharpoonup D\nabla V \quad 
\text{ weakly } \text{ in } L^2(\Omega _T). \end{aligned}$$

It follows from (40) that

$$\begin{aligned} T_{k/(k+1)}(D_k)^{1/2}\big (\nabla \widetilde{h}_k(D_k) + T_{k/(k+1)}(D_k)^{1/2}\nabla V_K\big )\rightharpoonup D\nabla h(D)+D\nabla V \end{aligned}$$

weakly in $$L^2(\Omega _T)$$.

Performing the limit $k\rightarrow \infty$ in the weak formulation (15)–(18), using formulation (25) of the fluxes, leads to the weak formulation of (1)–(4). The limit (*n*, *p*, *V*) satisfies the Dirichlet boundary conditions (6) since $n_k=\bar{n}$, $p_k=\bar{p}$, and $V_k=\bar{V}$ on $\Gamma _D$. Finally, the limit $n_k\rightharpoonup n$ weakly in $W^{1,7/5}(0,T;W_D^{1,2q/(q+4)}(\Omega )')\hookrightarrow C^0([0,T];W_D^{1,2q/(q+4)}(\Omega )')$ and the property $n_k(\cdot ,0)=n^I$ in the sense of $W_D^{1,2q/(q+4)}(\Omega )'$ show that *n* satisfies the initial condition in (5) in a weak sense. Similarly, *p* and *D* also satisfy the initial conditions.

### Energy Inequality

We identify the weak limit of $\nabla \widetilde{h}_k(D_k)$, which is needed in the limit of the energy inequality (34), leading to (14).

#### Lemma 17

Recall that $\widetilde{h}(s)=2\tanh ^{-1}\sqrt{s}-2\tanh ^{-1}\sqrt{1/2}$. It holds that

$$\begin{aligned} \nabla \widetilde{h}_k(D_k)\rightharpoonup \nabla \widetilde{h}(D) \quad \text{ weakly } \text{ in } L^2(\Omega _T). \end{aligned}$$

#### Proof

The function $\widetilde{h}_k$ is written explicitly as

$$\begin{aligned} \widetilde{h}_k(s) = {\left\{ \begin{array}{ll} 2\tanh ^{-1}\sqrt{s}-2\tanh ^{-1}\sqrt{1/2} & \text{ for } 0\le s\le \frac{k}{k+1}, \\ \sqrt{\frac{k+1}{k}}((k+1)s-k) + 2\tanh ^{-1}\sqrt{\frac{k}{k+1}} & \text{ for } s>\frac{k}{k+1}. \end{array}\right. } \end{aligned}$$

By Lemma 10, the sequence $(\nabla \widetilde{h}_k(D_k))$ is bounded in $L^2(\Omega _T)$. Proceeding as in the proof of Lemma 15, we can show that $(\widetilde{h}_k(D_k))$ is bounded in $L^2(0,T;H^1(\Omega ))$. We deduce from

$$\begin{aligned} |\nabla \widetilde{h}_k(D_{k,\eta })| \le {\left\{ \begin{array}{ll} |\nabla \widetilde{h}_k(D_k)| & \text{ for } D_{k,\eta }\le k/(k+1), \\ \sqrt{2}|\nabla L_k(D_{k,\eta })| & \text{ for } D_{k,\eta }>k/(k+1) \end{array}\right. } \end{aligned}$$

a uniform bound for $\nabla \widetilde{h}_k(D_{k,\eta })$ in $L^2(\Omega _T)$. The bound for $(\widetilde{h}_k(D_k))$ implies a bound for $(\widetilde{h}_k(D_{k,\eta }))$ in $L^2(\Omega _T)$, and we have $\widetilde{h}(D_\eta )\in L^2(\Omega _T)$. Finally, the proof of Lemma 16 shows that

$$\begin{aligned}&\widetilde{h}_k(D_k)\rightharpoonup 2\tanh ^{-1}\sqrt{D} - 2\tanh ^{-1}\sqrt{1/2}&\quad \text{ weakly } \text{ in } L^2(\Omega _T), \\&\nabla \widetilde{h}_k(D_k)\rightharpoonup 2\nabla \tanh ^{-1}\sqrt{D} \quad \text{ weakly } \text{ in } L^2(\Omega _T), \end{aligned}$$

ending the proof. $\square$

## Proof of Theorem 2

Since the proof is similar to that one of Jüngel and Vetter (2025, Theorem 2), we present only the key ideas, proceeding formally. First, we notice that Assumption (A5) with $r=3+\varepsilon >3$ yields

41 $$\begin{aligned} \Vert V\Vert _{L^\infty (0,T;W^{1,r}(\Omega ))} \le C\Vert n-p-D+A\Vert _{L^\infty (0,T;L^{3r/(r+3)}(\Omega ))} + C \le C, \end{aligned}$$

by Lemma 10, since $3r/(r+3)\le 5/3$ if $\varepsilon >0$ is sufficiently small. To simplify, we assume that $\bar{n}=0$. The proof can be extended in a straightforward way to general boundary data $\bar{n}$, but at the price of more elaborate technical estimations (see, e.g., Jüngel and Vetter 2025, Section 3). We wish to use $n^q$ for $q\in {{\mathbb {N}}}$ as a test function in the weak formulation of (1). To justify this step, we need to show that $n\in L^\infty (0,T;L^q(\Omega ))$. This property is proved in Jüngel and Vetter (2025, Sec. 3) in a similar context, and therefore, we do not present the quite technical proof here. With this test function, we obtain

$$\begin{aligned} \frac{1}{q+1}\frac{\textrm{d}}{\textrm{d}t}\int _\Omega n^{q+1}\textrm{d}x + q\int _\Omega ng'(n)n^{q-1}|\nabla n|^2 \textrm{d}x = q\int _\Omega n^q\nabla V\cdot \nabla n \textrm{d}x. \end{aligned}$$

We deduce from Lemma 20 that $ng'(n)\ge c(1+n^{2/3})$ and hence, using Hölder’s inequality for the drift term, integrating over (0, *t*), and possibly lowering the constant $c>0$,

42 $$\begin{aligned}&\frac{1}{q+1} \int _\Omega (n(t)^{q+1}-n(0)^{q+1})\textrm{d}x + \frac{c}{q+1}\int _0^t\int _\Omega \big (|\nabla n^{(q+1)/2}|^2 + |\nabla n^{(3q+5)/6}|^2\big )\textrm{d}x\textrm{d}s \nonumber \\&\quad \le \frac{6q}{3q+5}\int _\Omega n^{(3q+1)/6}\nabla V\cdot \nabla n^{(3q+5)/6} \textrm{d}x \nonumber \\&\quad \le C\int _0^t\Vert n^{(3q+1)/6}\Vert _{L^{(6+2\varepsilon )/(1+\varepsilon )}(\Omega )} \Vert \nabla V\Vert _{L^{3+\varepsilon }(\Omega )} \Vert \nabla n^{(3q+5)/6}\Vert _{L^2(\Omega )}\textrm{d}s \nonumber \\&\quad \le C\int _0^t\Vert n^{(3q+1)/6}\Vert _{L^{(6+2\varepsilon )/(1+\varepsilon )}(\Omega )} \Vert \nabla n^{(3q+5)/6}\Vert _{L^2(\Omega )}\textrm{d}s, \end{aligned}$$

where in the last step we have taken into account (41). We apply the Gagliardo–Nirenberg inequality with $\theta =(15+3\varepsilon )/(15+5\varepsilon )<1$ (at this point we need the regularity in $W^{1,r}(\Omega )$ with $r=3+\varepsilon >3$; $\varepsilon =0$ would not be sufficient) and Young’s inequality:

$$\begin{aligned} \Vert n^{(3q+1)/6}\Vert _{L^{(6+2\varepsilon )/(1+\varepsilon )}(\Omega )}&= \Vert n^{(3q+5)/6}\Vert _{L^{(3q+1)(6+2\varepsilon )/((3q+5)(1+\varepsilon ))} (\Omega )}^{(3q+1)/(3q+5)} \\&\le C + C\Vert n^{(3q+5)/6}\Vert _{L^{(3q+1)(6+2\varepsilon )/((3q+5)(1+\varepsilon ))} (\Omega )} \\&\le C + C\Vert \nabla n^{(3q+5)/6}\Vert _{L^2(\Omega )}^{\theta } \Vert n^{(3q+5)/6}\Vert _{L^1(\Omega )}^{1-\theta }. \end{aligned}$$

We insert this expression into (42), multiply by $q+1$, and use the Young inequality $(q+1)a^{1+\theta }b^{1-\theta }\le a^2 + C_\theta (q+1)^{2/(1-\theta )}b^2$, with $C_\theta >0$ depending solely on $\theta$:

$$\begin{aligned}&\Vert n(t)\Vert _{L^{q+1}(\Omega )}^{q+1} + c\int _0^t\Vert \nabla n^{(3q+5)/6}\Vert _{L^2(\Omega )}^2 \textrm{d}s \\&\quad \le \Vert n^I\Vert _{L^\infty (\Omega )}^{q+1} + C(q+1)\int _0^t\big (1+\Vert \nabla n^{(3q+5)/6}\Vert _{L^2(\Omega )}^{1+\theta } \Vert n^{(3q+5)/6}\Vert _{L^1(\Omega )}^{1-\theta }\big )\textrm{d}s \\&\quad \le \Vert n^I\Vert _{L^\infty (\Omega )}^{q+1} + CT(q+1) + \frac{c}{2}\int _0^t\Vert \nabla n^{(3q+5)/6}\Vert _{L^2(\Omega )}^2 \textrm{d}s \\&\qquad + C(q+1)^{2/(1-\theta )}\int _0^t\Vert n^{(3q+5)/6}\Vert _{L^1(\Omega )}^2 \textrm{d}s. \end{aligned}$$

The third term on the right-hand side can be absorbed by the left-hand side. Then, taking the supremum over (0, *T*) and observing that $\Vert n^{(3q+5)/6}\Vert _{L^1(\Omega )}^2 = \Vert n\Vert _{L^{(3q+5)/6}(\Omega )}^{(3q+5)/3}$:

43 $$\begin{aligned} \Vert n\Vert _{L^\infty (0,T;L^{q+1}(\Omega ))}^{q+1} \le \Vert n^I\Vert _{L^\infty (\Omega )}^{q+1} + C(q+1) + C(q+1)^{2/(1-\theta )}\Vert n\Vert _{L^\infty (0,T;L^{(3q+5)/6}(\Omega )) }^{(3q+5)/3}. \end{aligned}$$

We set $q_k:=q+1$ and $q_{k-1}:=(3q+5)/6$, which defines the recursion $q_{k-1}=(3q_k+2)/6$ or $q_k=2q_{k-1}-2/3$ with the explicit solution

$$\begin{aligned} q_k = 2^k\frac{3q_0-2}{3} + \frac{2}{3}, \quad k\in {{\mathbb {N}}}, \end{aligned}$$

where the initialization $q_0>2/3$ is arbitrary. Setting

$$\begin{aligned} b_k = \Vert n\Vert _{L^\infty (0,T;L^{q_k}(\Omega ))}^{q_k} + \Vert n^I\Vert _{L^\infty (\Omega )}^{q_k} + 1, \end{aligned}$$

an elementary computation shows that (43) can be written as

$$\begin{aligned} b_k \le C^k q_k^{2/(1-\theta )} b_{k-1}^2, \quad k\in {{\mathbb {N}}}. \end{aligned}$$

This inequality can be solved as in Jüngel and Vetter (2025, Sec. 3):

$$\begin{aligned} \Vert n\Vert _{L^\infty (0,T;L^{q_k}(\Omega ))} \le (C3^{2/(1-\theta )})^{(2^{k+1}-k-2)/q_k}\big ( \Vert n\Vert _{L^\infty (0,T;L^{q_0}(\Omega ))}^{q_0} + \Vert n^I\Vert _{L^\infty (\Omega )}^{q_0} + 1\big )^{2^k/q_k}. \end{aligned}$$

It is shown in Jüngel and Vetter (2025, Sec. 3) that the exponents on the right-hand side are bounded uniformly in *k*. Choosing $q_0\le 5/3$, it follows from Lemma 10 that $N:=\Vert n\Vert _{L^\infty (0,T;L^{q_0}(\Omega ))}$ is bounded. Then, the limit $k\rightarrow \infty$ shows that $\Vert n\Vert _{L^\infty (0,T;L^\infty (\Omega ))}\le C(n^I,N)$.

## Appendix A. Fermi–Dirac Integrals

The Fermi–Dirac integral of order $j>-1$ is defined by:

$$\begin{aligned} \mathcal {F}_j(z) = \frac{1}{\Gamma (j+1)}\int _0^\infty \frac{s^j}{1+e^{s-z}}\textrm{d}s, \quad z\in {{\mathbb {R}}}, \end{aligned}$$

where $\Gamma (j+1)=\int _0^\infty s^j e^{-s}\textrm{d}s$ is the Gamma function. This function has the property $\mathcal {F}_j'=\mathcal {F}_{j-1}$ for $j>0$. In the following, we write $A\sim B$ if there exist constants $C_1,C_2>0$ such that $A\le C_1B\le C_2 A$.

### Lemma 18

It holds for any $j>-1$ and $z\in {{\mathbb {R}}}$ that

$$\begin{aligned} \mathcal {F}_j(z) \sim e^z\textrm{1}_{\{z\le 0\}} + (z^{j+1}+1)\textrm{1}_{\{z>0\}}. \end{aligned}$$

### Proof

Let first $z\le 0$. Then $e^{s-z} < 1+e^{s-z}\le 2e^{s-z}$ and

$$\begin{aligned} \mathcal {F}_j(z) \ge \frac{1}{\Gamma (j+1)}\int _0^\infty \frac{s^j}{2e^{s-z}}\textrm{d}s =\frac{e^z}{2}, \quad \mathcal {F}_j(z) \le \frac{1}{\Gamma (j+1)}\int _0^\infty \frac{s^j}{e^{s-z}}\textrm{d}s = e ^z. \end{aligned}$$

This shows that $\mathcal {F}_j(z)\sim e^z$ for $z\le 0$. For $z>0$, we write $\mathcal {F}_j(z)=I_1+I_2$, where

$$\begin{aligned} I_1 = \frac{1}{\Gamma (j+1)}\int _0^z\frac{s^j}{1+e^{s-z}}\textrm{d}s, \quad I_2 = \frac{1}{\Gamma (j+1)}\int _z^\infty \frac{s^j}{1+e^{s-z}}\textrm{d}s. \end{aligned}$$

We infer from $s\le z$ and hence $e^{s-z}\le 1$ that

$$\begin{aligned} I_1&\ge \frac{1}{2\Gamma (j+1)}\int _0^z s^j \textrm{d}s = \frac{z^{j+1}}{2(j+1)\Gamma (j+1)} = \frac{z^{j+1}}{2\Gamma (j+2)}, \\ I_1&\le \frac{1}{\Gamma (j+1)}\int _0^z s^j \textrm{d}s = \frac{z^{j+1}}{\Gamma (j+2)}, \end{aligned}$$

To estimate $I_2$, we assume first that $j\ge 0$ and substitute $y=s-z\ge 0$:

$$\begin{aligned} I_2&= \frac{1}{\Gamma (j+1)}\int _0^\infty \frac{(y+z)^{j}}{1+e^y}\textrm{d}y \ge \frac{1}{\Gamma (j+1)}\int _0^\infty \frac{y^j}{2e^y}\textrm{d}y = \frac{1}{2}, \\ I_2&\le \frac{1}{\Gamma (j+1)}\bigg (\int _0^z+\int _z^\infty \bigg ) \frac{(y+z)^{j}}{1+e^y}\textrm{d}y \\&\le \frac{1}{\Gamma (j+1)}\int _0^z\frac{(2z)^j}{1+e^y}\textrm{d}y + \frac{1}{\Gamma (j+1)}\int _z^\infty \frac{(2y)^j}{1+e^y}\textrm{d}y \\&\le \frac{(2z)^j}{\Gamma (j+1)}\int _0^z e^{-y}\textrm{d}y + \frac{2^j}{\Gamma (j+1)}\int _z^\infty \frac{y^j}{e^y}\textrm{d}y \le \frac{(2z)^j}{\Gamma (j+1)} + 2^j. \end{aligned}$$

We conclude that

$$\begin{aligned} \mathcal {F}_j(z) \le \frac{z^{j+1}}{\Gamma (j+2)} + \frac{(2z)^j}{\Gamma (j+1)} + 2^j, \quad \mathcal {F}_j(z) \ge \frac{z^{j+1}}{2\Gamma (j+2)} + \frac{1}{2}, \end{aligned}$$

proving that $\mathcal {F}_j(z)\sim z^{j+1}+1$ for $z>0$ and $j\ge 0$.

Finally, let $-1<j<0$. Then, arguing as before, $I_1\le z^{j+1}/\Gamma (j+2)$,

$$\begin{aligned} I_2 = \frac{1}{\Gamma (j+1)}\int _0^\infty \frac{(y+z)^{j}}{1+e^y}\textrm{d}y \le \frac{1}{\Gamma (j+1)}\int _0^\infty \frac{y^{j}}{e^y}\textrm{d}y = 1, \end{aligned}$$

and hence $\mathcal {F}_j(z)\le z^{j+1}/\Gamma (j+2)+1$. The estimate from below requires a more careful computation. As the function $\mathcal {F}_j$ is continuous and strictly increasing, its minimum on [0, 1] equals $\mathcal {F}_j(0)$. Thus, for $z\in [0,1]$,

$$\begin{aligned} \mathcal {F}_j(z) \ge \mathcal {F}_j(0) = \frac{1}{\Gamma (j+1)}\int _0^\infty \frac{s^j}{1+e^s}\textrm{d}s > \frac{1}{\Gamma (j+1)}\int _0^\infty \frac{s^j}{2e^s}\textrm{d}s = \frac{1}{2}. \end{aligned}$$

Let $z>1$. We always have $I_2\ge 0$. The inequality $s^j\ge 1\ge z^{-1}$ for $1\le s\le z$ implies that $s^j\ge \frac{1}{2}(s^j+z^{-1})$ and hence,

$$\begin{aligned} I_1&= \frac{1}{\Gamma (j+1)}\bigg (\int _0^1+\int _1^z\bigg ) \frac{s^j}{1+e^{s-z}}\textrm{d}s \\&\ge \frac{1}{2\Gamma (j+1)}\int _0^1 s^j \textrm{d}s + \frac{1}{2\Gamma (j+1)}\int _1^z\frac{1}{2}(s^j + z^{-1})\textrm{d}s \\&= \frac{1}{4\Gamma (j+1)}\int _0^z s^j \textrm{d}s + \frac{1}{4\Gamma (j+1)}\int _0^1 s^j \textrm{d}s + \frac{1}{4\Gamma (j+1)}\int _1^z z^{-1}\textrm{d}s \\&= \frac{z^{j+1}}{4\Gamma (j+2)} + \frac{1}{4\Gamma (j+1)} \bigg (\frac{1}{j+1} + 1 - \frac{1}{z}\bigg ) \ge \frac{z^{j+1}}{4\Gamma (j+2)} + \frac{1}{4\Gamma (j+2)}. \end{aligned}$$

Combining the estimates for $z\in [0,1]$ and $z>1$ yields $\mathcal {F}_j(z)\ge C(z^{j+1}+1)$ for all $z\ge 0$. This is the desired lower bound, which concludes the proof. $\square$

### Corollary 19

Let $j>0$. Then for $z\in {{\mathbb {R}}}$,

$$\begin{aligned} \mathcal {F}_j'(z) = \mathcal {F}_{j-1}(z)\sim \mathcal {F}_j(z)\textrm{1}_{\{z\le 0\}} + \mathcal {F}_j(z)^{j/(j+1)}\textrm{1}_{\{z>0\}}. \end{aligned}$$

### Proof

If $z\le 0$, then, by Lemma 18, $\mathcal {F}_{j-1}(z)\sim e^z \sim \mathcal {F}_j(z)$. Furthermore, by the same lemma, if $z>0$, we have $\mathcal {F}_{j-1}(z)\sim z^j = (z^{j+1})^{j/(j+1)} \sim \mathcal {F}_j(z)^{j/(j+1)}$. $\square$

### Lemma 20

Recall that $g=\mathcal {F}_{1/2}^{-1}$. It holds for $z>0$ that

$$\begin{aligned} g'(z)\sim z^{-1} + z^{-1/3}. \end{aligned}$$

### Proof

Let $z>0$ and $y=\mathcal {F}_{1/2}^{-1}(z)$. Then, by Corollary 19,

$$\begin{aligned} g'(z)&= \frac{1}{\mathcal {F}'_{1/2}(y)} = \frac{1}{\mathcal {F}_{-1/2}(y)} \sim \frac{1}{\mathcal {F}_{1/2}(y)\textrm{1}_{\{y\le 0\}} + \mathcal {F}_{1/2}(y)^{1/3}\textrm{1}_{\{y>0\}}} \\&= \frac{1}{z\textrm{1}_{\{y\le 0\}} + z^{1/3}\textrm{1}_{\{y>0\}}} = z^{-1}\textrm{1}_{\{y\le 0\}} + z^{-1/3}\textrm{1}_{\{y>0\}}, \end{aligned}$$

which shows that $g'(z)\le C(z^{-1}+z^{-1/3})$ for $z>0$. For the lower bound, we distinguish the cases $0<z\le \mathcal {F}_{1/2}(0)$ (or $y\le 0$) and $z>\mathcal {F}_{1/2}(0)$ (or $y>0$). The case $z\le \mathcal {F}_{1/2}(0)$ implies that $z<1$ (since $1/2<\mathcal {F}_{1/2}(0)<1$). Then, the inequality $z^{-1}>z^{-1/3}$ yields $z^{-1}\textrm{1}_{\{y\le 0\}}>(z^{-1}+z^{-1/3})/2$ and $g'(z)\le C(z^{-1}+z^{-1/3})$.

Let $z>\mathcal {F}_{1/2}(0)$. If $z\ge 1$, we have $z^{-1/3}\textrm{1}_{\{y>0\}}\ge z^{-1}\textrm{1}_{\{y>0\}}$ and $g'(z)\ge C(z^{-1}+z^{-1/3})$. If $\mathcal {F}_{1/2}(0)<z<1$, we observe that $\mathcal {F}_{1/2}(0)<z$ yields $\mathcal {F}_{1/2}(0)z^{-1}<1<z^{-1/3}$, showing that

$$\begin{aligned} z^{-1/3}\textrm{1}_{\{y>0\}}> \frac{\mathcal {F}_{1/2}(0)}{2}(z^{-1}+z^{-1/3})\textrm{1}_{\{y>0\}} \end{aligned}$$

and again $g'(z)\ge C(z^{-1}+z^{-1/3})$. Summarizing these estimates, we conclude the proof. $\square$

Our final result is used in the proof of Lemma 13.

### Lemma 21

There exists a constant $C>0$ such that for $z>0$,

$$\begin{aligned} \frac{\textrm{d}}{\textrm{d}z}(zg'(z)) \le C\big (\textrm{1}_{\{z<\mathcal {F}_{1/2}(0)\}} + z^{-1/3}\textrm{1}_{\{z\ge \mathcal {F}_{1/2}(0)\}}\big ). \end{aligned}$$

### Proof

Let $z<\mathcal {F}_{1/2}(0)$ and set $y=\mathcal {F}_{1/2}^{-1}(z)<0$. Then

44 $$\begin{aligned} \frac{\textrm{d}}{\textrm{d}z}(zg'(z)) = g'(z) + zg''(z) = \frac{\mathcal {F}'_{1/2}(y)^2 - \mathcal {F}_{1/2}(y)\mathcal {F}_{1/2}''(y)}{\mathcal {F}'_{1/2}(y)^3}. \end{aligned}$$

Let $N(y):=\mathcal {F}'_{1/2}(y)^2 - \mathcal {F}_{1/2}(y)\mathcal {F}_{1/2}''(y)$. We compute the derivatives

$$\begin{aligned} \mathcal {F}'_{1/2}(z) = \frac{2}{\sqrt{\pi }}\int _0^\infty \frac{\sqrt{s} e^{s-z}}{(1+e^{s-z})^2}\textrm{d}s, \quad \mathcal {F}''_{1/2}(z) = \frac{2}{\sqrt{\pi }}\int _0^\infty \frac{\sqrt{s}e^{s-z}(e^{s-z}-1)}{(1+e^{s-z})^3}\textrm{d}s, \end{aligned}$$

yielding

$$\begin{aligned} N(y)&= \frac{4}{\pi }\int _0^\infty \int _0^\infty \frac{\sqrt{st}e^{s-y}e^{t-y}\textrm{d}s\textrm{d}t}{(1+e^{s-y})^2(1+e^{t-y})^2} - \frac{4}{\pi }\int _0^\infty \int _0^\infty \frac{\sqrt{st}e^{s-y}(e^{s-y}-1)\textrm{d}s\textrm{d}t}{(1+e^{s-y})^3(1+e^{t-y})} \\&= \frac{4}{\pi }\int _0^\infty \int _0^\infty \sqrt{st} e^{s-y} \frac{2e^{t-y}-e^{s-y}+1}{(1+e^{s-y})^3(1+e^{t-y})^2}\textrm{d}s\textrm{d}t \\&= \frac{4}{\pi }\int _0^\infty \int _0^\infty \sqrt{st}e^{s-y} \bigg (\frac{2}{(1+e^{s-y})^3(1+e^{t-y})} - \frac{1}{(1+e^{s-y})^2(1+e^{t-y})^2}\bigg )\textrm{d}s\textrm{d}t \\&= \frac{2}{\sqrt{\pi }}\mathcal {F}_{1/2}(y)\int _0^\infty \frac{2\sqrt{s} e^{s-y}}{(1+e^{s-y})^3}\textrm{d}s - \frac{2}{\sqrt{\pi }}\mathcal {F}_{1/2}'(y)\int _0^\infty \frac{\sqrt{t}}{(1+e^{t-y})^2}dt \\&\le \frac{2}{\sqrt{\pi }}\mathcal {F}_{1/2}(y)\int _0^\infty \frac{2\sqrt{s} e^{s-y}}{(1+e^{s-y})^3}\textrm{d}s. \end{aligned}$$

We estimate the remaining integral:

$$\begin{aligned} \frac{2}{\sqrt{\pi }}\int _0^\infty \frac{\sqrt{s} e^{s-y}}{(1+e^{s-y})^3}\textrm{d}s \le \frac{2}{\sqrt{\pi }}e^{y}\int _0^\infty \frac{\sqrt{s} e^{s-y}}{(1+e^{s-y})^2}\textrm{d}s = e^y\mathcal {F}'_{1/2}(y). \end{aligned}$$

Therefore, using Lemma 18 for $y=\mathcal {F}_{1/2}^{-1}(z)<0$,

$$\begin{aligned} N(y) \le 2e^y\mathcal {F}_{1/2}(y)\mathcal {F}'_{1/2}(y) \le C_1e^{3y}, \quad \mathcal {F}'_{1/2}(y)^3 = \mathcal {F}_{-1/2}(y)^3\ge C_2e^{3y}. \end{aligned}$$

We conclude from (44) for $z<\mathcal {F}_{1/2}(0)$ that

$$\begin{aligned} \frac{\textrm{d}}{\textrm{d}z}(zg'(z)) = \frac{N(y)}{\mathcal {F}_{1/2}'(y)^3} \le \frac{C_1}{C_2}. \end{aligned}$$

Next, let $z\ge \mathcal {F}_{1/2}(0)$ (or $y\ge 0$). We know from the proof of Lemma 20 that in this case $g'(z)\le Cz^{-1/3}$. According to (44), it remains to show that

$$\begin{aligned} zg''(z) = -\frac{\mathcal {F}_{1/2}(y)\mathcal {F}_{1/2}''(y)}{\mathcal {F}_{1/2}'(y)^3} \le Cz^{-1/3}\quad \text{ for } z\ge \mathcal {F}_{1/2}(0). \end{aligned}$$

To this end, we fix some $y_0>\mathcal {F}_{1/2}(0)$ and choose $0<\varepsilon <y_0$. For $y>y_0$, we split the integral $-\mathcal {F}''_{1/2}$ into two parts, $-\mathcal {F}''_{1/2}(y)=I_3+I_4$, where

$$\begin{aligned} I_3 = -\frac{2}{\sqrt{\pi }}\int _0^\varepsilon \frac{\sqrt{s}e^{s-y}(e^{s-y}-1)}{(1+e^{s-y})^3}\textrm{d}s, \quad I_4 = -\frac{2}{\sqrt{\pi }}\int _\varepsilon ^\infty \frac{\sqrt{s}e^{s-y}(e^{s-y}-1)}{(1+e^{s-y})^3}\textrm{d}s. \end{aligned}$$

The estimate of $I_3$ is straightforward:

$$\begin{aligned} I_3 \le \frac{2}{\sqrt{\pi }}e^{\varepsilon -y}\int _0^\varepsilon \sqrt{s}\textrm{d}s = \frac{4}{3\sqrt{\pi }}e^{\varepsilon -y}\varepsilon ^{3/2}. \end{aligned}$$

We integrate by parts in $I_4$ twice and split the resulting integral into two parts:

$$\begin{aligned} I_4&= \frac{2}{\sqrt{\pi }}\bigg ( \frac{\sqrt{s}e^{s-y}}{(1+e^{s-y})^2}\bigg |_\varepsilon ^\infty + \int _\varepsilon ^\infty \frac{s^{-1/2} e^{s-y}}{2(1+e^{s-y})^2}\textrm{d}s\bigg ) \\&= \frac{2}{\sqrt{\pi }}\bigg \{ -\frac{\sqrt{\varepsilon }e^{\varepsilon -y}}{(1+e^{\varepsilon -y})^2} + \bigg (\frac{s^{-1/2}}{2(1+e^{s-y})}\bigg |_\varepsilon ^\infty + \int _\varepsilon ^\infty \frac{s^{-3/2}}{4(1+e^{s-y})}\textrm{d}s\bigg )\bigg \} \\&= -\frac{2}{\sqrt{\pi }}\bigg ( \frac{\sqrt{\varepsilon }e^{\varepsilon -y}}{(1+e^{\varepsilon -y})^2} + \frac{\varepsilon ^{-1/2}}{2(1+e^{s-y})}\bigg ) + \frac{2}{\sqrt{\pi }}(I_5 + I_6), \end{aligned}$$

where

$$\begin{aligned} I_5&= \int _\varepsilon ^y\frac{s^{-3/2}}{4(1+e^{s-y})}\textrm{d}s \le \frac{1}{4(1+e^{\varepsilon -y})}\int _\varepsilon ^y s^{-3/2}\textrm{d}s = \frac{\varepsilon ^{-1/2}-y^{-1/2}}{2(1+e^{\varepsilon -y})}, \\ I_6&= \int _y^\infty \frac{s^{-3/2}}{4(1+e^{s-y})}\textrm{d}s \le \frac{1}{2}\int _y^\infty s^{-3/2}\textrm{d}s = y^{-1/2}. \end{aligned}$$

Summarizing these estimates, we observe that the contributions that are singular for $\varepsilon \rightarrow 0$ cancel, and we end up with

$$\begin{aligned} -\mathcal {F}_{1/2}''(y)&\le \frac{2}{\sqrt{\pi }}\bigg \{ \frac{2}{3} e^{\varepsilon -y}\varepsilon ^{3/2} - \bigg (\frac{\sqrt{\varepsilon }e^{\varepsilon -y}}{(1+e^{\varepsilon -y})^2} + \frac{\varepsilon ^{-1/2}}{2(1+e^{s-y})}\bigg ) + \frac{\varepsilon ^{-1/2}-y^{-1/2}}{2(1+e^{\varepsilon -y})} + y^{-1/2}\bigg \} \\&= \frac{2}{\sqrt{\pi }}\bigg ( \frac{2}{3} e^{\varepsilon -y}\varepsilon ^{3/2} - \frac{\sqrt{\varepsilon }e^{\varepsilon -y}}{(1+e^{\varepsilon -y})^2} + \frac{1+2e^{\varepsilon -y}}{2(1+e^{\varepsilon -y})}y^{-1/2}\bigg ). \end{aligned}$$

Since $\varepsilon >0$ is arbitrary, we can pass to the limit $\varepsilon \rightarrow 0$ to find that for all $y\ge 0$,

$$\begin{aligned} -\mathcal {F}''_{1/2}(y) \le \frac{2}{\sqrt{\pi }} \frac{1+2e^{-y}}{2(1+e^{-y})}y^{-1/2} \le C y^{-1/2}. \end{aligned}$$

We use the previous inequality to estimate the nominator and Corollary 19 to estimate the denominator in (44):

$$\begin{aligned} zg''(z) = -\frac{\mathcal {F}_{1/2}(y)\mathcal {F}''_{1/2}(y)}{\mathcal {F}_{1/2}'(y)^3} \le C\frac{\mathcal {F}_{1/2}(y)y^{-1/2}}{\mathcal {F}_{1/2}(y)} \le \frac{C}{y^{1/2}} \quad \text{ for } y>y_0. \end{aligned}$$

For $0\le y\le y_0$, the expression $zg''(z)$ is bounded since $\mathcal {F}_{1/2}$ and its derivatives are bounded in $[0,y_0]$. In case $y>y_0$, we wish to have an upper bound in terms of *z*. For this, we notice that, by Lemma 18, $z=\mathcal {F}_{1/2}(y)\le C_1(y^{3/2}+1)$. It follows from $y>y_0$ that $z\le C_1(1+y_0^{-3/2})y^{3/2}=:C_2 y^{3/2}$ and

$$\begin{aligned} zg''(z) \le Cy^{-1/2} \le CC_2^{1/3}z^{-1/3}. \end{aligned}$$

This concludes the proof. $\square$

## Appendix B. A Nonlinear Poincaré–Wirtinger-Type Lemma

We show a nonlinear version of the Poincaré–Wirtinger inequality.

### Lemma 22

Let $\Omega \subset {{\mathbb {R}}}^d$ ($d\ge 1$) be a bounded domain, let $f:[0,b)\rightarrow [0,\infty )$ with $b\in {{\mathbb {R}}}\cup \{+\infty \}$, be a strictly increasing function, let $u\in L^1(\Omega )$ satisfy $f(u)\in H^1(\Omega )$ and $u_\Omega :=\textrm{m}(\Omega )^{-1}\int _\Omega u\textrm{d}x<b$. Then for any $\widehat{u}\in (u_\Omega ,b)$ one has

$$\begin{aligned} \Vert f(u)\Vert _{L^2(\Omega )}^2\le 2m(\Omega )f(\widehat{u})^2 + 4C_P\bigg (1+\frac{\widehat{u}}{\widehat{u}-u_\Omega }\bigg ) \Vert \nabla f(u)\Vert _{L^2(\Omega )}^2, \end{aligned}$$

where $C_P>0$ is the square of the constant of the Poincaré–Wirtinger inequality.

Notice that the function *f* may be singular at *b*. Therefore, we need the condition $u_\Omega <b$ to ensure that the right-hand side is finite. We apply this lemma in the proof of Lemma 15 with $f(u)=-\log (1-u)$, $u\in {[0,1)}$.

### Proof

The proof is based on the arguments of (Cancès et al. 2021, Lemma 4.1). We set $A:=(f(u)-f(\widehat{u}))^+$ and $A_\Omega =\textrm{m}(\Omega )^{-1}\int _\Omega A\textrm{d}x$. Since $f(u)^2\le 2f(\widehat{u})^2 + 2[(f(u)-f(\widehat{u}))^+]^2 = 2f(\widehat{u})^2 + 2A^2$, we have

45 $$\begin{aligned} \int _\Omega f(u)^2 \textrm{d}x \le 4\int _\Omega (A-A_\Omega )^2 \textrm{d}x + 4\int _\Omega A_\Omega ^2 \textrm{d}x + 2\textrm{m}(\Omega )f(\widehat{u})^{2}. \end{aligned}$$

We set $C_1:=2\textrm{m}(\Omega )f(\widehat{u})^2$ for the last term. The first term is estimated according to the Poincaré–Wirtinger inequality as

46 $$\begin{aligned} \int _\Omega (A-A_\Omega )^2 \textrm{d}x \le C_P\Vert \nabla A\Vert _{L^2(\Omega )}^2 \le C_P\Vert \nabla f(u)\Vert _{L^2(\Omega )}^2. \end{aligned}$$

Furthermore,

$$\begin{aligned} \int _\Omega (A-A_\Omega )^2 \textrm{d}x = \int _{\{A=0\}}A_\Omega ^2 \textrm{d}x + \int _{\{A>0\}}(A-A_\Omega )^2 \textrm{d}x \ge \textrm{m}(\{A=0\})A_\Omega ^2. \end{aligned}$$

The previous two inequalities yield

47 $$\begin{aligned} A_\Omega ^2 \le \frac{C_P}{\textrm{m}(\{A=0\})}\Vert \nabla f(u)\Vert _{L^2(\Omega )}^2. \end{aligned}$$

We need to derive a lower bound for $\textrm{m}(\{A=0\})$. To this end, we observe that $A>0$ if and only if $u>\widehat{u}$ since *f* is assumed to be strictly increasing. Then,

$$\begin{aligned} \big (\textrm{m}(\Omega )-\textrm{m}(\{A=0\})\big )\widehat{u} = \textrm{m}(\{A>0\})\widehat{u} = \int _{\{u>\widehat{u}\}}\widehat{u}\textrm{d}x \le \int _\Omega u\textrm{d}x = \textrm{m}(\Omega )u_\Omega . \end{aligned}$$

It follows that

$$\begin{aligned} \textrm{m}(\{A=0\})\ge \textrm{m}(\Omega )\frac{\widehat{u}-u_\Omega }{\widehat{u}} > 0, \end{aligned}$$

and we infer from (47) that

$$\begin{aligned} A_\Omega ^2 \le \frac{\widehat{u}}{\widehat{u}-u_\Omega } \frac{C_P}{\textrm{m}(\Omega )}\Vert \nabla f(u)\Vert _{L^2(\Omega )}^2. \end{aligned}$$

Combining this inequality with (45) and (46), we obtain the result. $\square$
